# Supplementary material for: Changes in parental smoking during pregnancy and risks of adverse birth outcomes and childhood overweight in Europe and North America: An individual participant data meta-analysis of 229,000 singleton births
Source: PLoS Med. 2020 Aug 18;17(8):e1003182. doi: 10.1371/journal.pmed.1003182 (PMC7433860; doi:10.1371/journal.pmed.1003182)
Supplement: S1 Text. Protocol for analysis — (PDF) [file pmed.1003182.s002.pdf]

Parental smoking and risks of birth complications, childhood overweight and obesity: individual participant data meta-analysis

PROTOCOL FOR ANALYSIS

Background

While worldwide tobacco use is gradually declining among men, its use among women is rising: one in five women are expected to be tobacco users by 2025.(1) This trend is a cause for concern – particularly since smoking during pregnancy is considered a key avoidable risk factor for adverse pregnancy and birth outcomes, such as congenital abnormalities, stillbirth, sudden infant death syndrome, preterm birth and low birth weight.(2-9) Maternal smoking has also been linked to overweight, obesity and changes in body composition in childhood.(10, 11)

Despite what appears to be a clear link between maternal smoking and adverse child health, different studies into the effects of different smoking patterns during pregnancy have showed contradictory results. It is thought that assessing the different patterns of smoking during pregnancy could help to identify critical time windows. On the one hand, most studies have reported that children born to mothers who stopped smoking in early pregnancy do not have a higher risk of adverse birth outcomes, childhood overweight or obesity.(7, 8, 12-14) However, smoking only in early pregnancy has also been linked to fetal growth restriction and increased childhood BMI.(15-18) Little attention has been paid so far to dose-response relationships in the separate pregnancy trimesters and to the effects of a change in smoking habits during pregnancy. (12, 13, 19)

Child health may also be at risk when pregnant women are exposed to passive smoking, which can be measured using self-reported exposure, serum cotinine levels or paternal smoking. However, it is not yet clear whether exposure to passive smoking during pregnancy adversely affects birth outcomes.(12, 20-22) Paternal exposure is used not only for determining levels of passive smoking in the mother, but also for determining whether any associations found between maternal smoking and child health outcomes can be explained by direct intrauterine effects or simply reflect unmeasured confounders.(13) Direct intrauterine effects are assumed when effect estimates for maternal smoking with child health outcomes are stronger than effect estimates for paternal smoking, while similar effect estimates suggest that the associations are explained by unmeasured environmental or lifestyle factors. Previous studies show inconsistent results.(13, 23, 24)

To determine the associations between patterns of smoking during pregnancy and trimester-specific doses of maternal smoking on the one hand and birth outcomes and childhood BMI outcomes on the other, we will conduct an individual participant data meta-analysis among participants from pregnancy and birth cohort studies in America, Europe and Oceania. We also aim to determine the effects

of changes in maternal smoking habits and the effects of paternal smoking in non-smoking mothers on the aforementioned outcomes.

Methods

Inclusion criteria and participating cohorts

For this individual participant data meta-analysis we will use data from an existing collaboration on maternal obesity and childhood outcomes. Pregnancy & birth cohort studies participated if they included mothers with singleton live-born children born from 1989 onwards, had information available on maternal pre/early-pregnancy body mass index and at least one offspring measurement and were approved by their local institutional review boards. All cohort studies will provide written consent for using their data. Participants with no information on maternal smoking patterns and cohorts without variation in smoking patterns will be excluded from the analyses.

Parental smoke exposure

We will use trimester-specific maternal smoking information to categorize smoking during pregnancy in three groups (non-smoking; first trimester only smoking; continued smoking (as being any second or third trimester smoking)). Trimester-specific maternal smoking information will be categorized into non-smoking, ≤4 cigarettes/day, 5-9 cigarettes/day, and ≥10 cigarettes/day. We will combine the information about maternal smoking in first and third trimester to examine the change in smoking behavior. Information on paternal non-smoking/smoking will be used to explore the combined effects of maternal and paternal smoking. We combined the maternal and paternal smoking information into six categories: maternal and paternal non-smoking (used as reference category); maternal non-smoking and paternal smoking; maternal first trimester only smoking and paternal non-smoking; maternal first trimester only smoking and paternal smoking; maternal continued smoking and paternal non-smoking; and maternal continued smoking and paternal smoking.

Birth outcomes

We will create sex- and gestational age-adjusted birth weight standard deviation scores based on a North European reference chart.(25) Small size for gestational age at birth will be defined as sex- and gestational age-adjusted birth weight below the 10th percentile for each cohort specifically.

Childhood body mass index

We will create sex- and age-adjusted standard deviation scores (SDS) for early, mid- and late childhood BMI (2-5, >5-10 and >10-18 years, respectively) using WHO reference growth charts (Growth Analyzer

4.0, Dutch Growth Research Foundation).(26, 27) Childhood underweight, overweight and obesity will be defined using WHO cut offs.

Covariates

We will consider as potential confounders: child's sex, maternal age (years), educational level (low, medium, high), ethnicity (European/White, non-European/non-White), parity (nulliparous, multiparous), pre- or early pregnancy BMI (kg/m^2), paternal BMI (kg/m^2), alcohol consumption during pregnancy (yes, no), breastfeeding (never; <3 months; 3 - 6 months; ≥6 months), time of introduction of solid foods (<6 months; ≥6 months), television watching (≤2 hours/day; >2 hours/day), and paternal smoking (yes, no).

Statistical analysis

We will analyze the individual participant data from all cohorts simultaneously whilst accounting for clustering of participants within cohorts in multilevel model analyses.(28) First, we will use multilevel linear, binary and multinomial logistic mixed effects models to examine the associations of maternal smoking, explored across the trimesters of pregnancy and dose-response relationships within the first and third trimester separately, with birth outcomes and childhood BMI. Second, we will use similar models to investigate the associations of change in maternal smoking behavior between the first and third trimester of pregnancy with birth outcomes and childhood BMI. Third, we will use similar models to investigate the combined associations of maternal and paternal smoking with the risks of these outcomes. We will assess whether the risk estimates between categories statistically differed using the formula $Z = \frac{\beta 1 - \beta 2}{\sqrt{(SE\beta 1)^2 + (SE\beta 2)^2}}$.We will adjust all models potential confounders. All analyses focused on combined maternal and paternal smoking will be adjusted for the same covariates and paternal BMI. As sensitivity analyses, we will conduct a 2-stage random effects meta-analysis to study the same associations. To prevent exclusion of non-complete cases, for categorical variables missing values will be used as an additional group and in continuous variables missing values will be replaced by the median within the cohort or in the total variable if cohort specific measures are missing. We will conduct a sensitivity analysis with complete cases only. Also, to explore the influence on our results of using maternal age and BMI as categorical covariates, we will repeat the complete cases analysis using these covariates continuously. Statistical analyses will be performed using the Statistical Package of Social Sciences version 21.0 for Windows (SPSS Inc, Chicago, IL, USA).

REFERENCES

1. Samet JM, Yoon SY. Women and the tabacco epidemic: challenges for the 21st century: WHO, Institute for Global Tabacco Control, Johns Hopkins School of Public Health; 2011.
2. Cnattingius S. The epidemiology of smoking during pregnancy: smoking prevalence, maternal characteristics, and pregnancy outcomes. Nicotine Tob Res. 2004;6 Suppl 2:S125-40.
3. Hackshaw A, Rodeck C, Boniface S. Maternal smoking in pregnancy and birth defects: a systematic review based on 173 687 malformed cases and 11.7 million controls. Hum Reprod Update. 2011;17(5):589-604.
4. Marufu TC, Ahankari A, Coleman T, Lewis S. Maternal smoking and the risk of still birth: systematic review and meta-analysis. BMC Public Health. 2015;15:239.
5. Zhang K, Wang X. Maternal smoking and increased risk of sudden infant death syndrome: a meta-analysis. Leg Med (Tokyo). 2013;15(3):115-21.
6. Shah NR, Bracken MB. A systematic review and meta-analysis of prospective studies on the association between maternal cigarette smoking and preterm delivery. Am J Obstet Gynecol. 2000;182(2):465-72.
7. Raisanen S, Sankilampi U, Gissler M, Kramer MR, Hakulinen-Viitanen T, Saari J, et al. Smoking cessation in the first trimester reduces most obstetric risks, but not the risks of major congenital anomalies and admission to neonatal care: a population-based cohort study of 1,164,953 singleton pregnancies in Finland. J Epidemiol Community Health. 2014;68(2):159-64.
8. Moore E, Blatt K, Chen A, Van Hook J, DeFranco EA. Relationship of trimester-specific smoking patterns and risk of preterm birth. Am J Obstet Gynecol. 2016.
9. Kramer MS. Determinants of low birth weight: methodological assessment and meta-analysis. Bull World Health Organ. 1987;65(5):663-737.
10. Rayfield S, Plugge E. Systematic review and meta-analysis of the association between maternal smoking in pregnancy and childhood overweight and obesity. J Epidemiol Community Health. 2016.
11. Durmus B, Heppe DH, Taal HR, Manniesing R, Raat H, Hofman A, et al. Parental smoking during pregnancy and total and abdominal fat distribution in school-age children: the Generation R Study. Int J Obes (Lond). 2014;38(7):966-72.
12. Jaddoe VW, Troe EJ, Hofman A, Mackenbach JP, Moll HA, Steegers EA, et al. Active and passive maternal smoking during pregnancy and the risks of low birthweight and preterm birth: the Generation R Study. Paediatr Perinat Epidemiol. 2008;22(2):162-71.
13. Durmus B, Kruithof CJ, Gillman MH, Willemsen SP, Hofman A, Raat H, et al. Parental smoking during pregnancy, early growth, and risk of obesity in preschool children: the Generation R Study. Am J Clin Nutr. 2011;94(1):164-71.
14. Fasting MH, Oien T, Storro O, Nilsen TI, Johnsen R, Vik T. Maternal smoking cessation in early pregnancy and offspring weight status at four years of age. A prospective birth cohort study. Early Hum Dev. 2009;85(1):19-24.
15. Blatt K, Moore E, Chen A, Van Hook J, DeFranco EA. Association of reported trimester-specific smoking cessation with fetal growth restriction. Obstet Gynecol. 2015;125(6):1452-9.
16. Iniguez C, Ballester F, Costa O, Murcia M, Souto A, Santa-Marina L, et al. Maternal smoking during pregnancy and fetal biometry: the INMA Mother and Child Cohort Study. Am J Epidemiol. 2013;178(7):1067-75.
17. Grzeskowiak LE, Hodyl NA, Stark MJ, Morrison JL, Clifton VL. Association of early and late maternal smoking during pregnancy with offspring body mass index at 4 to 5 years of age. J Dev Orig Health Dis. 2015;6(6):485-92.
18. Mendez MA, Torrent M, Ferrer C, Ribas-Fito N, Sunyer J. Maternal smoking very early in pregnancy is related to child overweight at age 5-7 y. Am J Clin Nutr. 2008;87(6):1906-13.
19. Yan J, Groothuis PA. Timing of prenatal smoking cessation or reduction and infant birth weight: evidence from the United Kingdom Millennium Cohort Study. Matern Child Health J. 2015;19(3):447-58.

20. Windham GC, Eaton A, Hopkins B. Evidence for an association between environmental tobacco smoke exposure and birthweight: a meta-analysis and new data. Paediatr Perinat Epidemiol. 1999;13(1):35-57.
21. Inoue S, Naruse H, Yorifuji T, Kato T, Murakoshi T, Doi H, et al. Impact of maternal and paternal smoking on birth outcomes. J Public Health (Oxf). 2016.
22. Ahluwalia IB, Grummer-Strawn L, Scanlon KS. Exposure to environmental tobacco smoke and birth outcome: increased effects on pregnant women aged 30 years or older. Am J Epidemiol. 1997;146(1):42-7.
23. Howe LD, Matijasevich A, Tilling K, Brion MJ, Leary SD, Smith GD, et al. Maternal smoking during pregnancy and offspring trajectories of height and adiposity: comparing maternal and paternal associations. Int J Epidemiol. 2012;41(3):722-32.
24. Florath I, Kohler M, Weck MN, Brandt S, Rothenbacher D, Schottker B, et al. Association of pre- and post-natal parental smoking with offspring body mass index: an 8-year follow-up of a birth cohort. Pediatr Obes. 2014;9(2):121-34.
25. Niklasson A, Ericson A, Fryer JG, Karlberg J, Lawrence C, Karlberg P. An update of the Swedish reference standards for weight, length and head circumference at birth for given gestational age (1977-1981). Acta Paediatr Scand. 1991;90(8-9):756-62.
26. WHO Multicentre Growth Reference Study Group. WHO Child Growth Standards based on length/height, weight and age. Acta Paediatr Suppl. 2006;450:76-85.
27. de Onis M, Onyango AW, Borghi E, Siyam A, Nishida C, Siekmann J. Development of a WHO growth reference for school-aged children and adolescents. Bull World Health Organ. 2007;85(9):660-7.
28. Debray TP, Moons KG, Abo-Zaid GM, Koffijberg H, Riley RD. Individual participant data meta-analysis for a binary outcome: one-stage or two-stage? PLoS One. 2013;8(4):e60650.
