# Supplementary material for: Changes in parental smoking during pregnancy and risks of adverse birth outcomes and childhood overweight in Europe and North America: An individual participant data meta-analysis of 229,000 singleton births
Source: PLoS Med. 2020 Aug 18;17(8):e1003182. doi: 10.1371/journal.pmed.1003182 (PMC7433860; doi:10.1371/journal.pmed.1003182)
Supplement: S2 Text. Acknowledgments and funding — (PDF) [file pmed.1003182.s003.pdf]

S2 Text. Acknowledgments and funding

ACKNOWLEDGMENTS (PER COHORT)

ABCD

The authors especially thank all participating mothers and their children, and are grateful to all obstetric care providers in Amsterdam for their contribution to the data collection of the ABCD-study.

ALSPAC

The authors are extremely grateful to all the families who took part in this study, the midwives for their help in recruiting them, and the whole ALSPAC team, which includes interviewers, computer and laboratory technicians, clerical workers, research scientists, volunteers, managers, receptionists and nurses.

BAMSE

The authors thank all the children and their parents for participating in the BAMSE cohort and the nurses and other staff members working in the BAMSE project.

BIB

The authors acknowledge that Born in Bradford is only possible because of the enthusiasm and commitment of the children and parents in Born in Bradford. We are grateful to all participants, health professionals and researchers who have made Born in Bradford happen.

DNBC

The authors thank all the families for participating in the Danish National Birth Cohort.

EDEN

The authors thank the EDEN mother-child cohort study group, whose members are: I. Annesi-Maesano, J.Y. Bernard, J. Botton, M.A. Charles, P. Dargent-Molina, B. de Lauzon-Guillain, P. Ducimetière, M. de Agostini, B. Foliguet, A. Forhan, X. Fritel, A. Germa, V. Goua, R. Hankard, B. Heude, M. Kaminski, B. Larroque†, N. Lelong, J. Lepeule, G. Magnin, L. Marchand, C. Nabet, F Pierre, R. Slama, M.J. Saurel-Cubizolles, M. Schweitzer, O. Thiebaugeorges.

FCOU

The authors wish to acknowledge the University of Illinois at Chicago School of Public Health's Louise Hamilton Kyiv Data Management Center for their assistance in the data management for FCOU study.

GASPII

The authors acknowledge the families involved in the study.

Generation R

The authors gratefully acknowledge the contribution of all participants, general practitioners, hospitals, midwives, and pharmacies in Rotterdam.

Generation XXI

The authors gratefully acknowledge the families enrolled in Generation XXI for their kindness, all members of the research team for their enthusiasm and perseverance and the participating hospitals and their staff for their help and support.

GENESIS

The authors thank the Genesis research group which was comprised from Evdokia Oikonomou, Vivian Detopoulou, Christine Kortsalioudaki, Margarita Bartsota, Thodoris Liarigkovinos and Christos Vassilopoulos.

GINIplus

The authors thank all the families for their participation in the GINIplus study. Furthermore, the authors thank all members of the GINIplus Study Group for their excellent work. The GINIplus Study group consists of the following: Institute of Epidemiology I, Helmholtz Zentrum München, German Research Center for Environmental Health, Neuherberg (Heinrich J, Brüske I, Schulz H, Flexeder C, Zeller C, Standl M, Schnappinger M, Ferland M, Thiering E, Tiesler C); Department of Pediatrics, Marien-Hospital, Wesel (Berdel D, von Berg A); Ludwig-Maximilians-University of Munich, Dr von Hauner Children's Hospital (Koletzko S); Child and Adolescent Medicine, University Hospital rechts der Isar of the Technical University Munich (Bauer CP, Hoffmann U); IUF- Environmental Health Research Institute, Düsseldorf (Schikowski T, Link E, Klümper C, Krämer U, Sugiri D).

HUMIS

The authors thank the mothers who participated in the study and the Norwegian Research Council for their continuous support through several grants.

INMA-Valencia

The authors thank all the participants for their generous collaboration.

INMA-Gipuzkoa

The authors thank the children and parents who participated to the INMA-Gipuzkoa study.

INMA-Menorca

The authors thank all the participants for their generous collaboration. The authors are grateful to Mireia Garcia, Maria Victoria Estraña, Maria Victoria Iturriaga, Cristina Capo and Josep LLuch for their assistance in contacting the families and administering the questionnaires.

KOALA

The authors thank the children and parents who participated to the KOALA study.

LISAplus

The authors thank all the families for their participation in the LISAplus study. Furthermore, the authors thank all members of the LISAplus Study Group for their excellent work. The LISAplus Study group consists of the following: Helmholtz Zentrum München, German Research Center for Environmental Health, Institute of Epidemiology I, Munich (Heinrich J, Schnappinger M, Brüske I, Ferland M, Schulz H, Zeller C, Standl M, Thiering E, Tiesler C, Flexeder C); Department of Pediatrics, Municipal Hospital “St. Georg”, Leipzig (Borte M, Diez U, Dorn C, Braun E); Marien Hospital Wesel, Department of Pediatrics, Wesel (von Berg A, Berdel D, Stiers G, Maas B); Pediatric Practice, Bad Honnef (Schaaf B); Helmholtz Centre of Environmental Research – UFZ, Department of Environmental Immunology/Core Facility Studies, Leipzig (Lehmann I, Bauer M, Röder S, Schilde M, Nowak M, Herberth G , Müller J); Technical University Munich, Department of Pediatrics, Munich (Hoffmann U, Paschke M, Marra S); Clinical Research Group Molecular Dermatology, Department of Dermatology and Allergy, Technische Universität München (TUM), Munich (Ollert M, J. Grosch).

LUKAS

The authors thank all the families for their participation in the study. The authors are grateful to Raija Juntunen, Asko Vepsäläinen, Pekka Tiittanen, and Timo Kauppila for their contribution to the data collection and data management.

MoBa

The authors are grateful to all the participating families in Norway who take part in this on-going cohort study.

NINFEA

The authors thank all families participating in the NINFEA cohort.

PÉLAGIE

The authors thank the gynecologists, obstetricians, ultrasonographers, midwives, pediatricians, and families who participated in the study.

Piccolipiù

The authors acknowledge the Piccolipiù Working Group and the families involved in the study.

PRIDE Study

The authors thank the mothers and infants who participate in this ongoing cohort study, as well as all midwives, gynecologists, and general practitioners for their contributions to the data collection.

Project Viva

The authors thank the Project Viva mothers, children and families for their ongoing participation.

REPRO_PL

The authors would particularly like to thank all the cohort participants for their collaboration.

RHEA

The authors would particularly like to thank all the cohort participants for their generous collaboration.

SCOPE BASELINE

The authors are grateful to the families and healthcare staff who kindly gave their time to the study.

SWS

The authors are grateful to the women of Southampton who gave their time to take part in the Southampton Women's Survey and to the research nurses and other staff who collected and processed the data.

FUNDING (PER COHORT)

ABCD

This work was supported by the Netherlands Organization for Health Research and Development (ZonMw) (TOP grant, 40-00812-98-11010).

ALSPAC

The UK Medical Research Council and Wellcome (Grant ref: 102215/2/13/2) and the University of Bristol provide core support for ALSPAC. This study has received support from the US National Institute of Health (R01 DK10324) and European Research Council under the European Union's Seventh Framework Programme (FP7/2007-2013) / ERC grant agreement no 669545.

BAMSE

The Swedish Research Council, The Swedish Heart and Lung Foundation, The Swedish Research Council for Working Life and Social Welfare, the Swedish Asthma and Allergy Association Research Foundation, The Swedish Research Council Formas, Stockholm County Council, and the European Commission's Seventh Framework 29 Program MeDALL under grant agreement No. 261357.

BIB

BiB receives core infrastructure funding from the Wellcome Trust (WT101597MA) and a joint grant from the UK Medical Research Council (MRC) and Economic and Social Science Research Council (ESRC) (MR/N024397/1). This study has received support from the British Heart Foundation (CS/16/4/32482), US National Institute of Health (R01 DK10324) and European Research Council under the European Union's Seventh Framework Programme (FP7/2007-2013) / ERC grant agreement no 669545. The research was funded by the NIHR CLAHRC Yorkshire and Humber. www.clahrc-yh.nihr.ac.uk and

received support from the NIHR Clinical Research Network. The views expressed are those of the author(s), and not necessarily those of the NHS, the NIHR or the Department of Health and Social Care.

Co.N.ER

No funding reported.

DNBC

The Danish National Research Foundation has established the Danish Epidemiology Science Centre that initiated and created the Danish National Birth Cohort. The cohort is furthermore a result of a major grant from this foundation. Additional support for the Danish National Birth Cohort is obtained from the Pharmacy Foundation, the Egmont Foundation, the March of Dimes Birth Defects Foundation, the Augustinus Foundation, and the Health Foundation. The DNBC 7-year follow-up is supported by the Lundbeck Foundation (195/04) and the Danish Medical Research Council (SSVF 0646).

EDEN

The EDEN study was supported by: Foundation for medical research (FRM), National Agency for Research (ANR), National Institute for Research in Public health (IRESP: TGIR cohorte santé 2008 program), French Ministry of Health (DGS), French Ministry of Research, INSERM Bone and Joint Diseases National Research (PRO-A) and Human Nutrition National Research Programs, Paris-Sud University, Nestlé, French National Institute for Population Health Surveillance (InVS), French National Institute for Health Education (INPES), the European Union FP7 programmes (FP7/2007-2013, HELIX, ESCAPE, ENRIECO, Medall projects), Diabetes National Research Program (through a collaboration with the French Association of Diabetic Patients (AFD)), French Agency for Environmental Health Safety (now ANSES), Mutuelle Générale de l'Education Nationale a complementary health insurance (MGEN), French national agency for food security, French speaking association for the study of diabetes and metabolism (ALFEDIAM).

FCOU

FCOU study is supported by the U.S. National Institutes of Health Fogarty International Center, US NIEHS, US CDC, US EPA, and National Academy of Medical Sciences of Ukraine.

GASPII

Ministry of Health.

Generation R

The general design of the Generation R Study is made possible by financial support from the Erasmus MC, University Medical Center, Rotterdam, Erasmus University Rotterdam, Netherlands Organization for Health Research and Development (ZonMw), Netherlands Organisation for Scientific Research (NWO), Ministry of Health, Welfare and Sport and Ministry of Youth and Families. Research leading to these results has received funding from the European Union's Seventh Framework Programme (FP7/2007-2013), project EarlyNutrition under grant agreement n°289346, the European Union's Horizon 2020 research and innovation programme under grant agreement 733206 (LifeCycle Project).

Generation XXI

Generation XXI was funded by Programa Operacional de Saúde – Saúde XXI, Quadro Comunitário de Apoio III and Administração Regional de Saúde Norte (Regional Department of Ministry of Health). This study was funded by FEDER through the Operational Programme Competitiveness and Internationalization and national funding from the Foundation for Science and Technology – FCT (Portuguese Ministry of Science, Technology and Higher Education) (POCI-01- 0145-FEDER-016837), under the project "PathMOB.: Risco cardiometabólico na infância: desde o início da vida ao fim da infância" (Ref. FCT PTDC/DTP-EPI/3306/2014) and the Unidade de Investigação em Epidemiologia - Instituto de Saúde Pública da Universidade do Porto (EPIUnit) (POCI-01-0145-FEDER-006862; Ref. UID/DTP/04750/2013).

GENESIS

The study was supported by a research grant from Friesland Foods Hellas.

GINIplus

The GINIplus study was mainly supported for the first 3 years of the Federal Ministry for Education, Science, Research and Technology (interventional arm) and Helmholtz Zentrum Munich (former GSF) (observational arm). The 4 year, 6 year, 10 year and 15 year follow-up examinations of the GINIplus

study were covered from the respective budgets of the 5 study centres (Helmholtz Zentrum Munich (former GSF), Research Institute at Marien-Hospital Wesel, LMU Munich, TU Munich and from 6 years onwards also from IUF - Leibniz Research-Institute for Environmental Medicine at the University of Düsseldorf) and a grant from the Federal Ministry for Environment (IUF Düsseldorf, FKZ 20462296). Further, the 15 year follow-up examination of the GINIplus study was supported by the Commission of the European Communities, the 7th Framework Program: MeDALL project, and as well by the companies Mead Johnson and Nestlé.

HUMIS

European Community's Seventh Framework Programme (FP7/2007-2013) under grant agreements Early Nutrition n° 289346 and by funds from the Norwegian Research Council's MILPAAHEL programme, project No.213148.

INMA-Sabadell

This study was funded by grants from the Instituto de Salud Carlos III (Red INMA G03/176) and the Generalitat de Catalunya-CIRIT (1999SGR 00241).

INMA-Valencia

This study was funded by Grants from UE (FP7-ENV-2011 cod 282957 and HEALTH.2010.2.4.5-1), Spain: ISCIII (G03/176; FIS-FEDER: PI09/02647, PI11/01007, PI11/02591, PI11/02038, PI13/1944, PI13/2032, PI14/00891, PI14/01687, and PI16/1288; Miguel Servet-FEDER CP11/00178, CP15/00025, and CPII16/00051), and Generalitat Valenciana: FISABIO (UGP 15-230, UGP-15-244, and UGP-15-249).

INMA-Gipuzkoa

This study was funded by grants from the Instituto de Salud Carlos III (FISFIS PI06/0867, FIS-PS09/0009) 0867,Red INMA G03/176) and the Departamento de Salud del Gobierno Vasco (2005111093 and 2009111069) and the Provincial Government of Guipúzcoa (DFG06/004 and FG08/001).

INMA-Menorca

This study was funded by grants from the Instituto de Salud Carlos III (Red INMA G03/176).

KOALA

Data collection for the KOALA study was financially supported by unrestricted grants from: Royal Friesland Foods (Leeuwarden); Triodos Foundation (Zeist); Phoenix Foundation; Raphaël Foundation; Iona Foundation; Foundation for the Advancement of Heilpedagogie; the Netherlands Sugar Foundation (Baarn); Ministry of Economic Affairs; Netherlands Organisation for Health Research and Development (ZonMw 2100.0090); Netherlands Asthma Foundation (3.2.03.48 and 3.2.07.022); Stichting Astmabestrijding; Netherlands Heart Foundation (2008B112), all in the Netherlands.

LISAplus

The LISAplus study was mainly supported by grants from the Federal Ministry for Education, Science, Research and Technology and in addition from Helmholtz Zentrum Munich (former GSF), Helmholtz Centre for Environmental Research - UFZ, Leipzig, Research Institute at Marien-Hospital Wesel, Pediatric Practice, Bad Honnef for the first 2 years. The 4 year, 6 year, 10 year and 15 year follow-up examinations of the LISAplus study were covered from the respective budgets of the involved partners (Helmholtz Zentrum Munich (former GSF), Helmholtz Centre for Environmental Research - UFZ, Leipzig, Research Institute at Marien-Hospital Wesel, Pediatric Practice, Bad Honnef, IUF – Leibniz-Research Institute for Environmental Medicine at the University of Düsseldorf) and in addition by a grant from the Federal Ministry for Environment (IUF Düsseldorf, FKZ 20462296). Further, the 15 year follow-up examination of the LISAplus study was supported by the Commission of the European Communities, the 7th Framework Program: MeDALL project.

LUKAS

Grants from the Academy of Finland (grants 139021;287675); the Juho Vainio Foundation; the Foundation for Pediatric Research; EVO/VTR-funding; Päivikki and Sakari Sohlberg Foundation; The Finnish Cultural Foundation; European Union QLK4-CT-2001-00250; and by the National Institute for Health and Welfare, Finland.

MoBa

The Norwegian Mother and Child Cohort Study is supported by the Norwegian Ministry of Health and Care Services and the Ministry of Education and Research, NIH/NIEHS (contract no N01-ES-75558), NIH/NINDS (grant no.1 UO1 NS 047537-01 and grant no.2 UO1 NS 047537-06A1).

NINFEA

The NINFEA cohort was partially funded by the Compagnia San Paolo Fundation and by the Piedmont Region.

PÉLAGIE

The Pélagie cohort was supported by the French National Research Agency (ANR-2010-PRSP-007), and the French Research Institute for Public Health (AMC11004NSA-DGS).

Piccolipiù

The Piccolipiù project was financially supported by the Italian National Center for Disease Prevention and Control (CCM grants years 2010 and 2014) and by the Italian Ministry of Health (art 12 and 12 bis D.lgs 502/92).

PRIDE Study

The PRIDE Study is supported by grants from the Netherlands Organization for Health Research and Development, the Radboud Institute for Health Sciences, and the Lung Foundation Netherlands.

Project Viva

US National Institutes of Health (R01 HD034568, UH3 OD023286).

REPRO_PL

National Science Centre, Poland, under the grant DEC-2014/15/B/NZ7/00998, FP7 HEALS Grant N° 603946 and the Ministry of Science and Higher Education under grant agreement no. 3068/7.PR/2014/2.

RHEA

The "Rhea" project was financially supported by European projects (EU FP6-2003-Food-3-NewGeneris, EU FP6. STREP Hiwate, EU FP7 ENV.2007.1.2.2.2. Project No 211250 Escape, EU FP7-2008-ENV-1.2.1.4 Envirogenomarkers, EU FP7-HEALTH-2009- single stage CHICOS, EU FP7 ENV.2008.1.2.1.6. Proposal No 226285 ENRIECO, EU- FP7- HEALTH-2012 Proposal No 308333 HELIX) and the Greek

Ministry of Health (Program of Prevention of obesity and neurodevelopmental disorders in preschool children, in Heraklion district, Crete, Greece: 2011-2014; "Rhea Plus": Primary Prevention Program of Environmental Risk Factors for Reproductive Health, and Child Health: 2012-15).

SCOPE BASELINE

The SCOPE Ireland study was funded by the Irish Health Research Board. The Cork BASELINE Birth Cohort Study was funded by the National Children's Research Centre, Dublin, Ireland. SCOPE Ireland was funded by the Health Research Board, Ireland (CSA 2007/2). The BASELINE cohort was supported by the National Children's Research Centre, Dublin, Ireland, and the Food Standards Agency of the United Kingdom (grant no. TO7060). SCOPE and BASELINE are supported by INFANT, an SFI funded Research Centre (grant no 12/RC/2272).

SWS

The SWS is supported by grants from the Medical Research Council, National Institute for Health Research Southampton Biomedical Research Centre, University of Southampton and University Hospital Southampton National Health Service Foundation Trust, and the European Union's Seventh Framework Programme (FP7/2007-2013), project EarlyNutrition (grant 289346). Study participants were drawn from a cohort study funded by the Medical Research Council and the Dunhill Medical Trust.

ROLE OF THE FUNDING SOURCE

FCOU

Investigators from the U.S. National Institute of Environmental Health Scientists (NIEHS) were involved in the design of the birth outcomes phase of the FCOU study.

The other cohorts declared that the funding agencies had no role in the design and conduct of the study; collection, management, analysis and interpretation of data; preparation, review, approval of manuscript; or decision to submit manuscript for publication.
