# Supplementary material for: Changes in parental smoking during pregnancy and risks of adverse birth outcomes and childhood overweight in Europe and North America: An individual participant data meta-analysis of 229,000 singleton births
Source: PLoS Med. 2020 Aug 18;17(8):e1003182. doi: 10.1371/journal.pmed.1003182 (PMC7433860; doi:10.1371/journal.pmed.1003182)
Supplement: S1 Fig — (PDF) [file pmed.1003182.s004.pdf]

S1 Fig. Maternal continued smoking with risks of small size for gestational age assessed by 2-stage random effects models[a]

Continued smoking vs non-smoking

Study or Subgroup	Weight	Odds Ratio IV, Random, 95% CI	Odds Ratio IV, Random, 95% CI
BAMSE (Sweden)	11.2%	2.10 [1.56, 2.82]	
Co.N.ER (Italy)	2.2%	2.13 [0.96, 4.68]	
EDEN (France)	7.6%	1.66 [1.13, 2.44]	
GASPII (Italy)	2.1%	1.63 [0.73, 3.64]	
GENERATION R (The Netherlands)	18.5%	1.71 [1.41, 2.07]	
GENERATION XXI (Portugal)	19.6%	2.31 [1.93, 2.76]	
GENESIS (Greece)	9.2%	1.56 [1.11, 2.19]	
LUKAS (Finland)	1.5%	2.15 [0.81, 5.69]	
NINFEA (Italy)	3.8%	1.03 [0.58, 1.85]	
Piccolipiù (Italy)	9.0%	1.79 [1.27, 2.53]	
PRIDE Study (The Netherlands)	2.5%	2.02 [0.96, 4.21]	
Project Viva (United States)	3.7%	2.39 [1.33, 4.30]	
PÉLAGIE (France)	4.5%	1.47 [0.86, 2.49]	
REPRO_PL (Poland)	4.7%	2.97 [1.77, 4.97]	
**Total (95% CI)**	**100.0%**	**1.89 [1.67, 2.13]**	

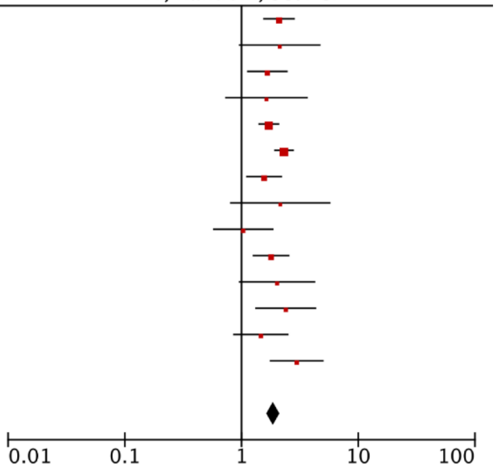

Heterogeneity: Tau^2 = 0.01; Chi^2 = 16.96, df = 13 (P = 0.20); I^2 = 23%
Test for overall effect: Z = 10.38 (P < 0.00001)

[a]Values are odds ratios (95% confidence intervals) per cohort and pooled from binary logistic regression models that reflect the risk of small size for gestational age for continued smoking compared to non-smoking. Models are adjusted for maternal age, educational level, parity, pre- or early pregnancy body mass index, alcohol consumption during pregnancy and paternal smoking. Analysis was restricted to cohorts with information on first trimester only smoking. The heterogeneity between the estimates of each cohort was 23% (95% CI 0%-65%).
