# Supplementary material for: Changes in parental smoking during pregnancy and risks of adverse birth outcomes and childhood overweight in Europe and North America: An individual participant data meta-analysis of 229,000 singleton births
Source: PLoS Med. 2020 Aug 18;17(8):e1003182. doi: 10.1371/journal.pmed.1003182 (PMC7433860; doi:10.1371/journal.pmed.1003182)
Supplement: S1 Table — (PDF) [file pmed.1003182.s005.pdf]

S1 Table. Cohort-specific methods of data collection for parental smoking, birth outcomes and childhood BMI

Cohort name, number of participants, birth years (country)	Maternal smoking	Paternal smoking	Gestational age at birth	Birth weight	Childhood weight and height
ABCD, n=7,324, 2003-2004 (The Netherlands)	Self-reported	NA	Clinical records	Clinical records	Measured
ALSPAC, n=12,148, 1991-1992 (United Kingdom)	Self-reported	Self-reported	Clinical records	Measured	Measured
BAMSE, n=4,057, 1994-1996 (Sweden)	Self-reported	Self-reported	Medical Birth Registry	Medical Birth Registry	Measured
BIB, n=1,641, 2007-2010 (United Kingdom)	Self-reported	NA	Clinical records	Clinical records	NA
Co.N.ER, n=641, 2004-2005 (Italy)	Self-reported	Self-reported	Self-reported	Clinical records	Self-reported
DNBC, n=71,710, 1996-2002 (Denmark)	Self-reported	Self-reported	Self-reported or National Medical Birth Registry	National Medical Birth Registry	Self-reported or measured
EDEN, n=1,880, 2003-2005 (France)	Self-reported	Self-reported	Clinical records	Clinical records	Measured or clinical records
FCOU, n=4,003, 1993-1996 (Ukraine)	Self-reported	Self-reported	NA	Clinical records	Clinical records
GASPII, n=680 (Italy)	Self-reported	Self-reported	Clinical records	Clinical records	Measured
GENERATION R, 7,934, 2002-2006 (The Netherlands)	Self-reported	Self-reported	Clinical records	Clinical records	Measured
GENERATION XXI, n=7,541, 2005-2006 (Portugal)	Self-reported	NA	Clinical records	Clinical records	Measured
GENESIS, n=2,261 , 2003-2004 (Greece)	Self-reported	NA	Clinical records	Clinical records	Measured
GINIplus, n=2,086 , 1995-1998 (Germany)	Self-reported	NA	NA	NA	Clinical records at 4y, measured and self-reported at 10 and 15y
HUMIS, n=986 , 2002-2009 (Norway)	Self-reported	NA	Clinical records	Clinical records	Self-reported
INMA, n=2,406, 1997-2008 (Spain)	Self-reported	Self-reported	Clinical records	Clinical records	Measured
KOALA, n=2,800, 2000-2002 (The Netherlands)	Self-reported	NA	Clinical records	Clinical records	Reported
LISAplus, n=1,965, 1997-1999 (Germany)	Self-reported	Self-reported	NA	NA	Clinical records at 4y, measured and self-reported at 10 and 15y
LUKAS, n=441, 2002-2005 (Finland)	Self-reported	NA	NA	Clinical records	Self-reported
MoBa, n=80,116, 1999-2009 (Norway)	Self-reported	Self-reported	Clinical records	Clinical records	Self-reported

NINFEA, n=2,259, 2005-2010 (Italy)	Self-reported	NA	Self-reported	Self-reported	Self-reported
PÉLAGIE, n=1,494, 2002-2005 (France)	Self-reported	Self-reported	Clinical records	Clinical records	NA
Piccolipiù, n=3,292, 2011-2015 (Italy)	Self-reported	Self-reported	Clinical records	Clinical records	NA
PRIDE Study, n=1,616, 2011-2015 (The Netherlands)	Self-reported	NA	Self-reported or clinical records	Self-reported or clinical records	NA
Project Viva, n=2,001, 1999-2002 (United States)	Self-reported	NA	Self-reported or clinical records	Clinical records	Measured
REPRO_PL, n=1,434, 2007-2011 (Poland)	Self-reported	Self-reported	Clinical records	Clinical records	Measured
RHEA, n=651, 2007-2008 (Greece)	Self-reported	Self-reported	Self-reported or clinical records	Clinical records	NA
SCOPE BASELINE, n=1,216, 2009-2011 (Ireland)	Self-reported	Self-reported	Measured	Measured	NA
SWS, n=2,716, 1998-2007 (United Kingdom)	Self-reported	NA	Measured	Clinical records	Measured

NA: Not available or not applicable. BMI, body mass index.
