# Supplementary material for: Changes in parental smoking during pregnancy and risks of adverse birth outcomes and childhood overweight in Europe and North America: An individual participant data meta-analysis of 229,000 singleton births
Source: PLoS Med. 2020 Aug 18;17(8):e1003182. doi: 10.1371/journal.pmed.1003182 (PMC7433860; doi:10.1371/journal.pmed.1003182)
Supplement: S2 Table — (PDF) [file pmed.1003182.s006.pdf]

S2 Table. Cohort-specific description of available covariates

**Cohort name, number of participants**	**Maternal age (years)**	**Maternal educational level**				**Maternal parity**		**Maternal pre- or early pregnancy BMI (kg/m²)**	**Paternal BMI (kg/m²)**	**Maternal alcohol consumption**	
		**Low**	**Medium**	**High**	**Missing**	**Nulliparous**	**Missing**			**Yes**	**Missing**
ABCD, n=7,324	31.0 (20.0, 40,0)	1547 (21.1)	2799 (38.2)	2916 (39.8)	62 (0.8)	4101 (56.0)	NA	22.2 (17.9, 33.8)	NA	1562 (21.3)	4 (0.1)
ALSPAC, n=12,148	28.0 (19.0, 38.0)	7132 (58.7)	2547 (21.0)	1506 (12.4)	963 (7.9)	5269 (43.4)	217 (1.8)	22.3 (17.9, 33.9)	24.9 (19.8, 32.7)	NA	12148 (100.0)
BAMSE, n=4,057	30.0 (22.0, 40.0)	1377 (33.9)	999 (24.6)	1655 (40.8)	26 (0.6)	2208 (54.4)	48 (1.2)	22.4 (18.2, 31.6)	NA	NA	4057 (100.0)
BIB, n=1,641	27.0 (17.0, 39.0)	412 (25.1)	609 (37.1)	614 (37.4)	6 (0.4)	624 (38.0)	24 (1.5)	24.7 (17.8, 39.8)	NA	308 (18.8)	7 (0.4)
Co.N.ER, n=641	33.7 (24.5, 41.9)	116 (18.1)	284 (44.3)	240 (37.4)	1 (0.2)	283 (44.1)	2 (0.3)	21.1 (17.6, 30.5)	24.8 (20.0, 31.1)	287 (44.8)	3 (0.5)
DNBC, n=71,710	30.3 (22.5, 39.2)	6625 (9.2)	27085 (37.8)	37742 (52.6)	258 (0.4)	34959 (48.8)	47 (0.1)	22.7 (18.0, 34.5)	24.8 (20.0, 32.4)	31754 (44.3)	79 (0.1)
EDEN, n=1,880	29.4 (20.3, 39.6)	531 (28.2)	334 (17.8)	1005 (53.5)	10 (0.5)	1039 (55.3)	3 (0.2)	22.1 (17.4, 34.6)	24.6 (19.3, 33.6)	944 (50.2)	101 (5.4)
FCOU, n=4,003	23.0 (17.0, 36.0)	242 (6.0)	2554 (63.8)	945 (23.6)	262 (6.5)	2594 (64.8)	307 (7.7)	21.7 (17.3, 31.6)	23.9 (19.6, 31.0)	837 (20.9)	193 (4.8)
GASPII, n=680	33.0 (22.0, 41.0)	96 (14.1)	341 (50.1)	243 (35.7)	NA	399 (58.7)	NA	21.3 (17.6, 31.2)	25.0 (20.7, 32.9)	240 (35.3)	3 (0.4)
GENERATION R, n=7,934	30.4 (19.3, 39.3)	821 (10.3)	3446 (43.4)	3280 (41.3)	387 (4.9)	4440 (56.0)	42 (0.5)	22.8 (18.0, 35.1)	24.9 (19.5, 33.0)	3642 (45.9)	700 (8.8)
GENERATION XXI, n=7,541	29.0 (18.0, 40.0)	2473 (32.8)	3282 (43.5)	1753 (23.2)	33 (0.4)	4213 (55.9)	95 (1.3)	22.9 (18.1, 34.7)	NA	847 (11.2)	364 (4.8)
GENESIS, n=2,261	30.4 (21.2, 39.2)	98 (4.3)	1135 (50.2)	947 (41.9)	81 (3.6)	1122 (49.6)	NA	21.9 (17.6, 31.1)	NA	108 (4.8)	NA
GINIplus, n=2,086	31.0 (24.0, 40.0)	243 (11.6)	874 (41.9)	964 (46.2)	5 (0.2)	NA	2086 (100.0)	22.1 (18.0, 31.6)	NA	NA	2086 (100.0)
HUMIS, n=986	30.0 (22.0, 39.0)	99 (10.0)	166 (16.8)	601 (61.0)	120 (12.2)	425 (43.1)	NA	23.4 (18.4, 35.1)	25.7 (21.0, 33.3)	72 (7.3)	400 (40.6)
INMA, n=2,406	30.0 (21.0, 39.0)	750 (31.2)	913 (37.9)	713 (29.6)	30 (1.2)	1305 (54.2)	2 (0.1)	22.5 (18.0, 34.9)	25.3 (20.3, 33.2)	219 (9.1)	29 (1.2)
KOALA, n=2,800	32.0 (25.0, 40.0)	286 (10.2)	1047 (37.4)	1328 (47.4)	139 (5.0)	1206 (43.1)	81 (2.9)	22.7 (18.4, 33.9)	NA	457 (16.3)	NA
LISAplus, n=1,965	32.0 (23.0, 40.0)	142 (7.2)	721 (36.7)	1089 (55.4)	13 (0.7)	855 (43.5)	7 (0.4)	21.7 (17.9, 32.8)	NA	1143 (58.2)	39 (2.0)
LUKAS, n=441	30.8 (21.2, 42.1)	21 (4.8)	334 (75.7)	86 (19.5)	NA	151 (34.2)	NA	24.1 (18.5, 36.4)	26.1 (20.1, 34.7)	NA	441 (100.0)

S2 Table. Cohort-specific description of available covariates (continued)

Cohort name, number of participants	Maternal age (years)	Maternal educational level				Maternal parity		Maternal pre- or early pregnancy BMI (kg/m^2)	Paternal BMI (kg/m^2)	Maternal alcohol consumption	
		Low	Medium	High	Missing	Nulliparous	Missing			Yes	Missing
MoBa, n=80,116	30.0 (21.0, 39.0)	25127 (31.4)	32959 (41.1)	20428 (25.5)	1602 (2.0)	36477 (45.5)	NA	23.1 (18.4, 34.9)	25.4 (20.5, 33.4)	10259 (12.8)	607 (0.8)
NINFEA, n=2,259[a]	33.0 (25.0, 41.0)	92 (4.1)	774 (34.3)	1388 (61.4)	5 (0.2)	1519 (67.2)	2 (0.1)	21.5 (17.4, 32.0)	24.5 (19.7, 31.7)	895 (39.6)	5 (0.2)
PÉLAGIE, n=1,353	30.2 (22.8, 39.6)	190 (14.0)	238 (17.6)	992 (68.1)	3 (0.2)	591 (43.7)	5 (0.4)	21.6 (17.7, 32.2)	NA	202 (14.9)	13 (1.0)
Piccolipiù, n=3,292	34.0 (23.0, 42.0)	393 (11.9)	1415 (43.0)	1473 (44.7)	11 (0.3)	1916 (58.2)	8 (0.2)	21.7 (17.6, 33.0)	24.8 (20.2, 32.7)	1452 (44.1)	37 (1.1)
PRIDE Study, n=1,616	30.0 (24.0, 38.0)	31 (1.9)	329 (20.4)	1219 (75.4)	37 (2.3)	973 (60.2)	5 (0.3)	22.6 (18.4, 33.5)	24.1 (19.4, 31.2)	300 (18.6)	219 (13.6)
Project Viva, n=2,001	32.3 (19.5, 40.9)	662 (33.1)	724 (36.2)	603 (30.1)	12 (0.6)	959 (47.9)	NA	23.4 (18.1, 39.2)	25.9 (20.0, 35.2)	1245 (62.2)	137 (6.8)
REPRO_PL, n=1,434	28.0 (20.0, 37.0)	171 (11.9)	429 (29.9)	820 (57.2)	14 (1.0)	804 (56.1)	13 (0.9)	21.5 (17.3, 31.2)	NA	116 (8.1)	108 (7.5)
RHEA, n=651	30.0 (20.0, 40.0)	109 (16.7)	322 (49.5)	213 (32.7)	7 (1.1)	NA	651 (100.0)	23.4 (18.1, 36.1)	26.8 (21.2, 36.3)	126 (19.4)	201 (30.9)
SCOPE BASELINE, n=1,216	31.0 (20.0, 39.0)	NA	162 (13.3)	1045 (85.9)	9 (0.7)	1216 (100.0)	NA	23.9 (19.1, 34.9)	26.5 (20.9, 33.9)	431 (35.4)	36 (3.0)
SWS, n=2,716	30.2 (22.8, 36.3)	334 (12.3)	1594 (58.7)	782 (28.8)	6 (0.2)	1339 (49.3)	2 (0.1)	24.2 (18.9, 37.5)	25.5 (19.8, 34.1)	2093 (77.1)	254 (9.4)
**Total group**	30.0 (20.3, 39.2)	50120 (21.9)	88416 (38.6)	86520 (37.8)	4102 (1.8)	110987 (48.4)	3647 (1.6)	22.7 (18.1, 34.6)	25.1 (20.1, 33.1)	59539 (26.0)	22271 (9.7)

Values are expressed as number of participants (%) or medians (95% range). BMI, body mass index.

[a]Subset of participants with follow-up completed at 4 years of child's age by the time of data transfer (March 2015).
