# Supplementary material for: Changes in parental smoking during pregnancy and risks of adverse birth outcomes and childhood overweight in Europe and North America: An individual participant data meta-analysis of 229,000 singleton births
Source: PLoS Med. 2020 Aug 18;17(8):e1003182. doi: 10.1371/journal.pmed.1003182 (PMC7433860; doi:10.1371/journal.pmed.1003182)
Supplement: S3 Table — (PDF) [file pmed.1003182.s007.pdf]

S3 Table. Cohort-specific description of maternal smoking variables

Cohort name, number of participants	Maternal first trimester smoking		Categories (cigarettes/day)			Maternal third trimester smoking		Categories (cigarettes/day)		
	No	Yes	<1-4	5-9	≥10	No	Yes	<1-4	5-9	≥10
ABCD, n=7,324[a]	5451 (90.8)	550 (9.2)	NA	NA	NA	4439 (90.1)	490 (9.9)	253 (3.6)	118 (1.7)	94 (1.3)
ALSPAC, n=12,148	9687 (79.7)	2461 (20.3)	404 (3.4)	559 (4.7)	1473 (12.3)	9581 (78.9)	2567 (21.1)	498 (4.1)	622 (5.1)	1394 (11.5)
BAMSE, n=4,057	3574 (88.1)	483 (11.9)	100 (2.5)	114 (2.8)	267 (6.7)	3671 (90.5)	386 (9.5)	109 (2.7)	87 (2.1)	188 (4.6)
BIB, n=1,641	1418 (86.4)	223 (13.6)	NA	NA	NA	NA	NA	NA	NA	NA
Co.N.ER, n=641	555 (86.6)	86 (13.4)	48 (7.6)	26 (4.1)	12 (1.9)	582 (90.8)	59 (9.2)	29 (4.5)	19 (3.0)	11 (1.7)
DNBC, n=71,710	59316 (82.7)	12391 (17.3)	2351 (3.3)	4103 (5.7)	5896 (8.3)	55659 (81.4)	12680 (18.6)	1631 (2.3)	3885 (5.5)	6066 (8.6)
EDEN, n=1,880	1397 (74.5)	478 (25.5)	109 (5.8)	160 (8.6)	208 (11.2)	1544 (83.2)	312 (16.8)	95 (5.1)	111 (5.9)	98 (5.3)
FCOU, n=4,003	3647 (91.1)	356 (8.9)	223 (5.6)	91 (2.3)	41 (1.0)	3647 (91.1)	356 (8.9)	223 (5.6)	91 (2.3)	41 (1.0)
GASPII, n=680	603 (88.7)	77 (11.3)	45 (6.7)	16 (2.4)	16 (2.4)	627 (92.2)	53 (7.8)	28 (4.1)	14 (2.1)	11 (1.6)
GENERATION R, n=7,934	5662 (79.0)	1503 (21.0)	799 (10.4)	401 (5.2)	303 (3.9)	5596 (84.6)	1020 (15.4)	506 (6.5)	321 (4.1)	193 (2.5)
GENERATION XXI, n=7,541	5796 (76.9)	1739 (23.1)	NA	NA	NA	6405 (85.0)	1129 (15.0)	NA	NA	NA
GENESIS, n=2,261	1894 (83.8)	367 (16.2)	150 (6.8)	116 (5.3)	100 (4.5)	1897 (83.9)	364 (16.1)	158 (7.0)	114 (5.0)	92 (4.1)
GINIplus, n=2,086	1903 (91.8)	171 (8.2)	40 (1.9)	58 (2.8)	72 (3.5)	1903 (92.0)	165 (8.0)	69 (3.3)	59 (2.7)	38 (1.8)
HUMIS, n=986	935 (94.8)	51 (5.2)	6 (0.6)	18 (1.8)	22 (2.2)	759 (93.4)	54 (6.6)	11 (1.1)	17 (1.7)	16 (1.6)
INMA, n=2,406	1992 (82.8)	414 (17.2)	127 (5.3)	116 (4.8)	166 (6.9)	1978 (82.6)	418 (17.4)	169 (7.0)	149 (6.2)	100 (4.2)
KOALA, n=2,800	2616 (93.4)	184 (6.6)	32 (1.2)	82 (3.0)	70 (2.5)	2594 (92.6)	206 (7.4)	42 (1.5)	67 (2.4)	97 (3.5)
LISAplus, n=1,965	1713 (87.2)	252 (12.8)	85 (4.4)	73 (3.8)	90 (4.6)	1743 (91.6)	160 (8.4)	54 (2.8)	53 (2.7)	47 (2.4)
LUKAS, n=441	371 (84.1)	70 (15.8)	NA	NA	NA	407 (92.3)	34 (7.7)	NA	NA	NA
MoBa, n=80,116[a]	72315 (91.1)	7090 (8.9)	NA	NA	NA	51108 (92.0)	4428 (8.0)	1970 (2.5)	1327 (1.7)	1084 (1.4)
NINFEA, n=2,259[b]	2092 (92.6)	167 (7.4)	NA	NA	NA	2129 (94.7)	120 (5.3)	NA	NA	NA
PÉLAGIE, n=1,494	1025 (75.8)	328 (24.2)	119 (8.8)	108 (8.0)	97 (7.2)	1160 (89.2)	140 (10.8)	72 (6.2)	47 (4.1)	15 (1.3)
Piccolipiù, n=3,292	2584 (78.5)	707 (21.5)	458 (14.0)	156 (4.8)	93 (2.8)	2983 (90.6)	308 (9.4)	177 (5.4)	81 (2.5)	46 (1.4)
PRIDE Study, n=1,616	1531 (94.9)	82 (5.1)	40 (2.5)	22 (1.4)	19 (1.2)	1402 (97.1)	42 (2.9)	17 (1.1)	11 (0.7)	9 (0.6)
Project Viva, n=2,001	1744 (90.1)	192 (9.9)	NA	NA	NA	1880 (96.4)	70 (3.6)	NA	NA	NA
REPRO_PL, n=1,434	1224 (85.7)	205 (14.3)	52 (3.8)	57 (4.1)	58 (4.2)	1296 (92.0)	113 (8.0)	34 (2.4)	36 (2.5)	36 (2.5)
RHEA, n=651	545 (84.5)	100 (15.5)	29 (4.7)	23 (3.7)	23 (3.7)	NA	NA	NA	NA	NA
SCOPE BASELINE, n=1,216	1079 (88.7)	137 (11.3)	63 (5.2)	40 (3.3)	24 (2.0)	NA	NA	NA	NA	NA
SWS, n=2,716	2350	358	61	102	195	2155	400	75	96	228

	(86.8)	(13.2)	(2.3)	(3.8)	(7.3)	(84.3)	(15.7)	(2.8)	(3.5)	(8.4)
**Total group**	117253 (83.3)	23582 (16.7)	5341 (2.5)	6441 (3.0)	9245 (4.3)	167145 (86.5)	26074 (13.5)	6220 (2.8)	7322 (3.3)	9904 (4.4)

Values are expressed as number of participants (valid %). NA, not available or not applicable. Maternal first trimester smoking refers to all maternal smoking in first trimester no matter if mothers stop or continue smoking after first trimester.

[a]Numbers of smoking (no/yes) in first trimester are second trimester smoking numbers. To prevent exclusion due to no information on first trimester smoking, nonsmokers from second trimester were used as nonsmoking during pregnancy (ABCD and MoBa).

[b]Subset of participants with follow-up completed at 4 years of child's age by the time of data transfer (March 2015). Data on number of cigarettes/day smoked in pregnancy were not used for this manuscript.
