# Supplementary material for: Changes in parental smoking during pregnancy and risks of adverse birth outcomes and childhood overweight in Europe and North America: An individual participant data meta-analysis of 229,000 singleton births
Source: PLoS Med. 2020 Aug 18;17(8):e1003182. doi: 10.1371/journal.pmed.1003182 (PMC7433860; doi:10.1371/journal.pmed.1003182)
Supplement: S4 Table — BMI, body mass index. (PDF) [file pmed.1003182.s008.pdf]

S4 Table. Associations of maternal smoking with gestational age at birth, birth weight and childhood BMI

	Gestational age at birth in weeks (95% Confidence Interval)	Gestational age-adjusted birth weight SDS (95% Confidence Interval)	Childhood BMI SDS (95% Confidence Interval)
**No maternal smoking**	*Reference* n=188357	*Reference* n=190873	*Reference* n=92434
**Only first trimester smoking**	0.06 (-0.01, 0.14) n=2116	0.02 (-0.02, 0.06) n=2144	0.04 (-0.02, 0.10) n=1084
**First trimester dosage**			
≤4 cigarettes/day	0.09 (-0.03, 0.21) n=828	0.04 (-0.03, 0.11) n=826	-0.05 (-0.16, 0.06) n=340
5-9 cigarettes/day	0.04 (-0.16, 0.24) n=288	0.02 (-0.09, 0.13) n=288	0.05 (-0.13, 0.22) n=136
≥10 cigarettes/day	-0.02 (-0.22, 0.19) n=273	0.09 (-0.02, 0.21) n=271	0.06 (-0.11, 0.22) n=152
**Continued smoking**	-0.05 (-0.07, -0.03)** n=29951	-0.37 (-0.38, -0.36)** n=30125	0.19 (0.17, 0.21)** n=13083
**Continued smoking dosage**			
≤4 cigarettes/day	-0.08 (-0.12, -0.03)* n=5866	-0.22 (-0.25, -0.20)** n=6034	0.16 (0.12, 0.20)** n=2792
5-9 cigarettes/day	-0.11 (-0.15, -0.07)** n=7115	-0.43 (-0.46, -0.41)** n=7162	0.18 (0.14, 0.21)** n=3284
≥10 cigarettes/day	-0.15 (-0.19, -0.12)** n=9771	-0.55 (-0.57, -0.53)** n=9743	0.23 (0.19, 0.26)** n=4139

Values are beta's (95% confidence intervals) from multilevel linear mixed effects models that reflect the differences in gestational age at birth in weeks, gestational age-adjusted birth weight in standard deviation scores and childhood body mass index in standard deviation scores per smoking group compared with the reference group (no maternal smoking).
Number of cigarettes used as continued smoking dosage were based on third trimester information.
Models are adjusted for maternal age, educational level, parity, pre- or early pregnancy body mass index, alcohol consumption during pregnancy and paternal smoking. *P-value<0.05; **P-value<0.001.
BMI, body mass index; SDS, standard deviation score.
