# Supplementary material for: Changes in parental smoking during pregnancy and risks of adverse birth outcomes and childhood overweight in Europe and North America: An individual participant data meta-analysis of 229,000 singleton births
Source: PLoS Med. 2020 Aug 18;17(8):e1003182. doi: 10.1371/journal.pmed.1003182 (PMC7433860; doi:10.1371/journal.pmed.1003182)
Supplement: S5 Table — BMI, body mass index. (PDF) [file pmed.1003182.s009.pdf]

S5 Table. Change in maternal smoking habits during pregnancy, gestational age at birth, birth weight and childhood BMI

	Gestational age at birth in weeks (95% Confidence Interval)	Gestational age-adjusted birth weight SDS (95% Confidence Interval)	Childhood BMI SDS (95% Confidence Interval)
**No maternal smoking in first trimester**			
Third trimester no smoking	*Reference* n=100634	*Reference* n=103740	*Reference* n=59070
Third trimester ≤4 cigarettes/day	-0.04 (-0.28, 0.16) n=278	-0.10 (-0.22, 0.01) n=274	0.25 (0.09, 0.42)* n=147
Third trimester 5-9 cigarettes/day	0.07 (-0.25, 0.39) n=104	-0.28 (-0.46, -0.09)* n=103	0.18 (-0.09, 0.46) n=51
Third trimester ≥10 cigarettes/day	0.03 (-0.34, 0.40) n=80	-0.35 (-0.56, -0.14)* n=79	0.19 (-0.16, 0.54) n=31
**Maternal smoking in first trimester ≤4 cigarettes/day**			
Third trimester quitted	0.09 (-0.03, 0.20) n=862	-0.01 (-0.08, 0.06) n=859	0.01 (-0.09, 0.11) n=388
Third trimester ≤4 cigarettes/day	0.02 (-0.05, 0.09) n=2261	-0.23 (-0.27, -0.20)** n=2457	0.17 (0.11, 0.23)** n=1169
Third trimester 5-9 cigarettes/day	-0.10 (-0.21, 0.02) n=885	-0.40 (-0.46, -0.33)** n=880	0.26 (0.16, 0.35)** n=440
Third trimester ≥10 cigarettes/day	-0.21 (-0.46, 0.03) n=186	-0.46 (-0.60, -0.32)** n=185	0.20 (-0.01, 0.41) n=86
**Maternal smoking in first trimester 5-9 cigarettes/day**			
Third trimester quitted	0.04 (-0.16, 0.23) n=304	-0.06 (-0.17, 0.05) n=304	0.06 (-0.09, 0.22) n=165
Third trimester ≤4 cigarettes/day	0.03 (-0.10, 0.16) n=657	-0.28 (-0.36, -0.21)** n=654	0.21 (0.10, 0.33)** n=307
Third trimester 5-9 cigarettes/day	-0.05 (-0.11, 0.01) n=3551	-0.42 (-0.45, -0.39)** n=3617	0.17 (0.12, 0.22)** n=1704
Third trimester ≥10 cigarettes/day	-0.10 (-0.20, -0.01)* n=1330	-0.51 (-0.56, -0.46)** n=1319	0.20 (0.13, 0.28)** n=632
**Maternal smoking in first trimester ≥10 cigarettes/day**			
Third trimester quitted	0.02 (-0.18, 0.22) n=285	0.03 (-0.08, 0.14) n=283	0.13 (-0.02, 0.27) n=194
Third trimester ≤4 cigarettes/day	-0.05 (-0.22, 0.13) n=358	-0.24 (-0.34, -0.14)* n=354	0.16 (0.01, 0.30)* n=192
Third trimester 5-9 cigarettes/day	-0.12 (-0.22, -0.02)*	-0.51 (-0.57, -0.45)**	0.18 (0.09, 0.27)**

	n=1078	n=1072	n=503
Third trimester ≥10 cigarettes/day	-0.15 (-0.20, -0.11)**	-0.55 (-0.58, 0.53)**	0.25 (0.22, 0.07)**

Values are beta's (95% confidence intervals) from multilevel linear mixed effects models that reflect the differences in gestational age at birth in weeks, gestational age-adjusted birth weight in standard deviation scores and childhood BMI in standard deviation scores per smoking group compared with the reference group (non-smoking in first and third trimester). Models are adjusted for maternal age, educational level, parity, pre-pregnancy body mass index, alcohol consumption during pregnancy and paternal smoking. *P-value<0.05; **P-value<0.001. BMI, body mass index; SDS, standard deviation score.
