# Supplementary material for: Changes in parental smoking during pregnancy and risks of adverse birth outcomes and childhood overweight in Europe and North America: An individual participant data meta-analysis of 229,000 singleton births
Source: PLoS Med. 2020 Aug 18;17(8):e1003182. doi: 10.1371/journal.pmed.1003182 (PMC7433860; doi:10.1371/journal.pmed.1003182)
Supplement: S6 Table — BMI, body mass index. (PDF) [file pmed.1003182.s010.pdf]

S6 Table. Associations of maternal and paternal smoking with gestational age at birth, birth weight and childhood BMI

	Gestational age at birth in weeks (95% Confidence Interval)	Gestational age-adjusted birth weight SDS (95% Confidence Interval)	Childhood BMI SDS (95% Confidence Interval)
**Maternal non-smoking**			
Paternal non-smoking	*Reference* n=123666	*Reference* n=123328	*Reference* n=59395
Paternal smoking	-0.03 (-0.05, -0.01)* n=31890	-0.01 (-0.02, 0.00) n=33691	0.06 (0.04, 0.08)** n=15474
**Maternal first trimester smoking**			
Paternal non-smoking	0.09 (-0.08, 0.26) n=412	0.04 (-0.06, 0.13) n=412	0.01 (-0.12, 0.14) n=233
Paternal smoking	0.03 (-0.10, 0.17) n=626	0.04 (-0.04, 0.11) n=625	0.04 (-0.08, 0.15) n=305
**Maternal continued smoking**			
Paternal non-smoking	-0.03 (-0.07, 0.00) n=8768	-0.34 (-0.36, -0.32)** n=8723	0.14 (0.10, 0.17)** n=3872
Paternal smoking	-0.08 (-0.11, -0.06)** n=15806	-0.42 (-0.44, -0.40)** n=15967	0.26 (0.23, 0.28)** n=6661

Values are beta's (95% confidence intervals) from multilevel linear mixed effects models that reflect the differences in gestational age at birth in weeks, gestational age-adjusted birth weight in standard deviation scores and childhood BMI in standard deviation scores per smoking group compared with the reference group (no parental smoking).
Models are adjusted for maternal age, maternal BMI, paternal BMI, maternal education, parity and maternal alcohol consumption during pregnancy.
*P-value<0.05; **P-value<0.001. BMI, body mass index; SDS, standard deviation score.
