# Supplementary material for: Changes in parental smoking during pregnancy and risks of adverse birth outcomes and childhood overweight in Europe and North America: An individual participant data meta-analysis of 229,000 singleton births
Source: PLoS Med. 2020 Aug 18;17(8):e1003182. doi: 10.1371/journal.pmed.1003182 (PMC7433860; doi:10.1371/journal.pmed.1003182)
Supplement: S7 Table — BMI, body mass index. (PDF) [file pmed.1003182.s011.pdf]

S7 Table. Complete cases analysis of maternal smoking with risks of birth complications and childhood overweight (with maternal age and BMI in categories)

	**Preterm birth Odds Ratio (95% Confidence Interval)**	**Small size for gestational age at birth Odds Ratio (95% Confidence Interval)**	**Childhood overweight Odds Ratio (95% Confidence Interval)**
**No maternal smoking**	*Reference* $n_{cases/total}$ =5859/138839	*Reference* $n_{cases/total}$ =12340/140086	*Reference* $n_{cases/total}$ = 10932/63318
**Only first trimester smoking**	0.76 (0.53, 1.09) $n_{cases/total}$ =33/941	0.92 (0.73, 1.17) $n_{cases/total}$ =82/941	1.13 (0.89, 1.43) $n_{cases/total}$ =102/473
**Continued smoking**	1.02 (0.94, 1.10) $n_{cases/total}$ =980/20943	2.16 (2.06, 2.26)** $n_{cases/total}$ =3621/21068	1.41 (1.33, 1.50)** $n_{cases/total}$ =2110/8667

Values are odds ratios (95% confidence intervals) from multilevel binary logistic mixed effects models that reflect the risk of preterm birth, small size for gestational age and childhood overweight per smoking group compared with the reference group (no maternal smoking).
Preterm birth is defined as birth before the gestational age of 37 weeks. Small size for gestational age is defined as the lowest 10% of sex and gestational age adjusted birth weight standard deviation score per cohort. Childhood overweight is overweight and obesity together according to the World Health Organization criteria. Models are adjusted for maternal age, educational level, parity, pre- or early pregnancy BMI, alcohol consumption during pregnancy and paternal smoking, all categorized. *P-value<0.05; **P-value<0.001. BMI, body mass index.
