# Supplementary material for: Changes in parental smoking during pregnancy and risks of adverse birth outcomes and childhood overweight in Europe and North America: An individual participant data meta-analysis of 229,000 singleton births
Source: PLoS Med. 2020 Aug 18;17(8):e1003182. doi: 10.1371/journal.pmed.1003182 (PMC7433860; doi:10.1371/journal.pmed.1003182)
Supplement: S9 Table — (PDF) [file pmed.1003182.s013.pdf]

## S9 Table. Contact information for data requests per cohort

| Cohort name (country) | Contact for data requests | Local institutional ethical review boards |
|---|---|---|
| ABCD (The Netherlands) | abcd@amc.nl | Central Committee on Research Involving Human Subjects in The Netherlands, the medical ethics review committees of the participating hospitals and the Registration Committee of the Municipality of Amsterdam |
| ALSPAC (United Kingdom) | http://www.bristol.ac.uk/alspac/researchers/ | ALSPAC Ethics and Law Committee and Local Research Ethics Committees |
| BAMSE (Sweden) | bamse-projekt@imm.ki.se | Regional ethical review board in Stockholm (application numbers 2010/1474- 31/3 and 2013/1879- 32). |
| BIB (United Kingdom) | borninbradford@bthft.nhs.uk | Bradford Research Ethics Comittee |
| Co.N.ER (Italy) | simona.rosa@unibo.it | Ethics Committee of the S. Orsola-Malpighi Teaching Hospital of Bologna (Italy) (052/2004/U/Tess) |
| DNBC (Denmark) | dnbc-research@ssi.dk | The Scientific Ethic Committee in Denmark, the Danish Data Protection Agency, and the DNBC Steering Committee |
| EDEN (France) | etude.eden@inserm.fr | Ethics Committee of the Bicêtre Hospital |
| FCOU (Ukraine) | zoreslava7@ukr.net | Institutional Review Boards at the University of Illinois at Chicago and the Ukrainian Institute for Pediatrics, Obstetrics, and Gynecology |
| GASPII (Italy) | p.lorusso@deplazio.it | Ethical Committee of the Università Cattolica del Sacro Cuore, Rome |
| Generation R (The Netherlands) | generationr@erasmusmc.nl | Medical Ethical Committee of the Erasmus Medical Center, Rotterdam |
| Generation XXI (Portugal) | gxxi@med.up.pt | Ethics Committee of Hospital de S. João |
| GENESIS (Greece) | oandrou@hua.gr | Ethical Committee of Harokopio University of Athens and all municipalities invited to participate in the study |
| GINIplus (Germany) | maike.ferland@helmholtz-muenchen.de | Bavarian General Medical Council, University of Leipzig, Medical Council of North-Rhine-Westphalia |
| HUMIS (Norway) | ingunn.brandt@fhi.no | Regional Ethics Committee for Medical Research in Norway (reference S-02122) and Norwegian Data Inspectorate |
| INMA (Spain) | inma@proyectoimna.org | The Municipal Institute of Sanitary Assistance of Barcelona, La Fe University Hospital of Valencia, The Donostia Hospital, and Ib-salut |
| KOALA (The Netherlands) | datahub@maastrichtuniversity.nl | Medical ethics committee of the Maastricht University/University Hospital of Maastricht |
| LISAplus (Germany) | maike.ferland@helmholtz-muenchen.de | Bavarian General Medical Council, University of Leipzig, Medical Council of North-Rhine-Westphalia |
| LUKAS (Finland) | pirkka.kirjavainen@thl.fi | Research Ethics Committee, Hospital District of Northern Savo, Kuopio, Finland |
| MoBa (Norway) | datatilgang@fhi.no | Norwegian Data Inspectorate and the Regional Ethics Committee for Medical Research |
| NINFEA (Italy) | info@progettoninfea.it | Ethical Committee of the San Giovanni Battista Hospital and CTO/CRF/Maria Adelaide Hospital of Turin (approval N.0048362 and following amendments) |
| PÉLAGIE (France) | pelagie.rennes@inserm.fr | French Consulting Committee for the Treatment of Information in Medical Research (no. 09.485) and the French National Commission for the Confidentiality of Computerised Data (no. 909347) |

| | | |
|---|---|---|
| Piccolipiù (Italy) | piccolipiuroma@deplazio.it | Ethics committees of the Local Health Unit Roma E (management centre), of the Istituto Superiore di Sanità (National Institute of Public Health) and of each local centre |
| PRIDE Study (The Netherlands) | info@pridestudy.nl | Regional Committee on Research involving Human Subjects |
| Project Viva (United States) | project_viva@hphc.org | Institutional Review Board of Harvard Pilgrim Health Care |
| REPRO_PL (Poland) | impx@imp.lodz.pl | Ethical Committee of the Nofer Institute of Occupational Medicine, Łódź, Poland (Decision No. 7/2007) |
| RHEA (Greece) | kliniki.diatrofis@med.uoc.gr | Ethical Committee of the University Hospital, Scientific Council, Heraklion, Crete, Greece |
| SCOPE BASELINE (Ireland) | epvchls@liverpool.ac.uk baseline@ucc.ie | Clinical Research Ethics Committee of the Cork Teaching Hospitals, [ref ECM5(9) 01/07/ 2008] |
| SWS (United Kingdom) | sws@mrc.soton.ac.uk | Southampton and South West Hampshire Local Research Ethics Committee (06/Q1702/104) |
