# Supplementary material for: Changes in parental smoking during pregnancy and risks of adverse birth outcomes and childhood overweight in Europe and North America: An individual participant data meta-analysis of 229,000 singleton births
Source: PLoS Med. 2020 Aug 18;17(8):e1003182. doi: 10.1371/journal.pmed.1003182 (PMC7433860; doi:10.1371/journal.pmed.1003182)
Supplement: Attachment — Submitted filename: philips.pdf [file pmed.1003182.s014.pdf]

This paper describes an individual participant data meta-analysis (IPD) of the relationship between paternal smoking and child outcomes.

I was asked for a statistical report and I interpret that to include all aspects of the design and conduct of the study.

Points of detail

Page 5 Just for the record I think using 230000 in the title is fine even though strictly speaking there are only 229158. If anyone objects I would replace it with 220000+ rather than the exact number.

Page 7 The authors have not claimed that this is a systematic review intending to cover all known studies. However the process of selection is not clear, at least to me, and we could use a bit more detail.

Page 7 The countries represented are all in Europe apart from one study from the United States. Apart from Ukraine they all seem to be what the World Bank calls high–income. Is this an artefact of the selection method presented here? Does the focus primarily on high income countries affect generalisability?

Page 7 and 8 Most of the variables have been categorised. As I am sure the authors know categorising an essentially continuous variable wastes information (Altman and Royston, 2006; Royston et al., 2006) and leads to models which are often implausible as they predict the effect remaining flat within categories and then jumping to a new value at the category boundary. I can see why in an IPD study this is sometimes inevitable as primary studies used incompatible classifications (in which case perhaps say so) but why categorise the anthropometric measures which presumable were recorded in grams and centimetres everywhere.

Page 10 Confidence intervals for I^2 would also be helpful to reveal our uncertainty about the heterogeneity. I know we did not use to use them but I am persuaded by Ioannidis et al. (2007) that we should. I am rather surprised by the low levels of heterogeneity seen in the supplementary figures. These are not gigantic samples but they are all fairly substantial and following the arguments in Rücker et al. (2008) I would have expected more heterogeneity.

Page 10 and Table 3 I think the smaller numbers in Table 3 might justify more caution in interpreting any which fail to reach some level of statistical significance.

Page 10 The interactions presented here have been given as estimates for the separate strata and a p–value for the test but we do not have an estimate for the difference or interaction. If the authors have tested for interactions formally then why not present the relative odds ratio so that we can see how much one is greater than the other with a confidence interval?

Page 32 Table 1 Of the three outcomes this shows us the prevalence of childhood overweight but not the prevalence of pre–term birth or of small for gestational age. Since they are what is being analysed that would seem helpful information to add.

Page 32 Table 1 In the footnote here what exactly does 'selected samples' mean? Are these clinical samples with restricted ranges on some variables?

Point of more substance

The 2–stage estimates presented in the supplement are useful because they give estimates of heterogeneity and also the important information that not all studies contribute to all estimates. Unless I have missed this it seems important information to be included in the main report since the sample on which first trimester only versus none is being compared is not the same sample as the continued versus none. The plots are fine in the supplement but they are under–played in the main text I think.

Summary

Mostly requests for more information and points to clarify.

Michael Dewey

References

D G Altman and P Royston. The cost of dichotomising continuous variables. *British Medical Journal*, 332:1080, 2006.

J Ioannidis, N Patsopoulos, and E Evangelou. Uncertainty in heterogeneity estimates in meta–analyses. *British Medical Journal*, 335:914–916, 2007.

P Royston, D G Altman, and W Sauerbrei. Dichotomizing continuous predictors in multiple regression: a bad idea. *Statistics in Medicine*, 25:127–141, 2006.

G Rücker, G Schwarzer, J R Carpenter, and M Schumacher. Undue reliance on I^2 in assessing heterogeneity may mislead. *BMC Medical Research Methodology*, 8(79), 2008.
