# Supplementary material for: Changes in parental smoking during pregnancy and risks of adverse birth outcomes and childhood overweight in Europe and North America: An individual participant data meta-analysis of 229,000 singleton births
Source: PLoS Med. 2020 Aug 18;17(8):e1003182. doi: 10.1371/journal.pmed.1003182 (PMC7433860; doi:10.1371/journal.pmed.1003182)
Supplement: Attachment — Submitted filename: response letter.docx [file pmed.1003182.s015.docx]

**Response to the editors and reviewers**

In this document, you will find our specific responses to all the questions or comments from the editors and reviewers, including the corresponding changes in the manuscript. Textual changes, which are part of the revised manuscript, are underlined.

**Editors**

**Comment 1**

Please remove all language that implies causality, throughout your manuscript. Reference should be made to associations instead.

***Response:*** *We have* *removed all language that implies causality throughout the manuscript.*

**Comment 2**

Please cite reference numbers in square brackets, leaving a space before the reference bracket, and removing spaces between reference numbers where more than one reference is cited.

***Response:*** *We have performed the corresponding changes in the manuscript.*

**Comment 3**

Please rename supplementary figures/tables as Supplementary Figure / Table, etc rather than eFigure and eTable

***Response:*** *We have renamed the supplementary tables and figures according to PLOS Medicine´s guidelines.*

**Comment 4**

Please revise your title according to PLOS Medicine's style, placing the study design in the subtitle (ie, after a colon). We suggest “Changes in parental smoking during pregnancy and risks of adverse birth outcomes and childhood overweight in Europe and North America: an individual participant data meta-analysis of 229,000 singleton births” or similar.

***Response:*** *We have changed the title to* ***´****Changes in parental smoking during pregnancy and risks of adverse birth outcomes and childhood overweight in Europe and North America: an individual participant data meta-analysis of 229,000 singleton births´ (Page 1).*

**Comment 5**

Data Availability Statement: PLOS Medicine requires that the de-identified data underlying the specific results in a published article be made available, without restrictions on access, in a public repository or as Supporting Information at the time of article publication, provided it is legal and ethical to do so. Please see the policy at <https://eur01.safelinks.protection.outlook.com/?url=http%3A%2F%2Fjournals.plos.org%2Fplosmedicine%2Fs%2Fdata-availability&amp;data=02%7C01%7Cv.jaddoe%40erasmusmc.nl%7C244ad0d7cfef4b65e27b08d7bea83d18%7C526638ba6af34b0fa532a1a511f4ac80%7C0%7C0%7C637187502100435363&amp;sdata=9xVi7TUELlTUU4p6Pi%2FLHabeiCnWtGDbuDv0g7oWOjE%3D&amp;reserved=0> and FAQs at

<https://eur01.safelinks.protection.outlook.com/?url=http%3A%2F%2Fjournals.plos.org%2Fplosmedicine%2Fs%2Fdata-availability%23loc-faqs-for-data-policy&amp;data=02%7C01%7Cv.jaddoe%40erasmusmc.nl%7C244ad0d7cfef4b65e27b08d7bea83d18%7C526638ba6af34b0fa532a1a511f4ac80%7C0%7C0%7C637187502100435363&amp;sdata=AzzlOnxU%2F4fwpiOy2IU8U2aPSDg47CBL8%2BAB4%2FRn2Hc%3D&amp;reserved=0>

Please provide appropriate contact(s) (web or email address) to whom requests for access to de-identified data can be made. Please note that this cannot be a study author.

***Response:*** *We have provided the Data Availability Statement in the online submission as follows: ´Data from 28 different cohorts with different data publishing policies were used. The datasets generated and analyzed for this study are available upon request to the executive committees of the individual cohorts. Contacts for data requests for each cohort are listed in S9 Table.´*

**Comment 6**

Please remove the ‘Research in context’ section, replacing instead with a short, non-technical Author Summary of your research to make findings accessible to a wide audience that includes both scientists and non-scientists. The Author Summary should immediately follow the Abstract in your revised manuscript. This text is subject to editorial change and should use non-identical language, that is distinct from the scientific abstract. Please see our author guidelines for more information: <https://eur01.safelinks.protection.outlook.com/?url=https%3A%2F%2Fjournals.plos.org%2Fplosmedicine%2Fs%2Frevising-your-manuscript%23loc-author-summary&amp;data=02%7C01%7Cv.jaddoe%40erasmusmc.nl%7C244ad0d7cfef4b65e27b08d7bea83d18%7C526638ba6af34b0fa532a1a511f4ac80%7C0%7C0%7C637187502100435363&amp;sdata=aksgzxj2d9JFDGBj2qSR1ynif9uJz4Qriu4vba4WFtk%3D&amp;reserved=0>

***Response:*** *We have replaced the ´Research in context´ Section by an ´Author Summary´, as follows (Pages 6 and 7):*

***´Author summary***

***Why was this study done?***

- *Maternal smoking during pregnancy is an important risk factor for various birth complications and childhood overweight.*
- *It is not clear whether this increased risk is also present if mothers smoke during the first trimester only or reduce the number of cigarettes during pregnancy.*
- *The associations of paternal smoking with birth and childhood outcomes also remain unknown.*

***What did the researchers do and find?***

- *We conducted an individual participant data meta-analysis using data from* *229,158 families from 28 pregnancy and birth cohorts from Europe and North America to assess the associations of parental smoking during pregnancy, specifically of quitting or reducing smoking and maternal and paternal smoking combined, with preterm birth, small size for gestational age and childhood overweight.*
- *We observed that smoking in the first trimester only did not increase the risk of preterm birth and small size for gestational age but was associated with a higher risk of childhood overweight, as compared to non-smoking. Reducing the number of cigarettes during pregnancy, without quitting, was still associated with higher risks of these adverse outcomes.*
- *Paternal smoking seems to be associated, independently of maternal smoking, with the risks of childhood overweight.*

***What do these findings mean?***

- *Population strategies should focus on parental smoking prevention before or at the start, rather than, during pregnancy.*
- *Future studies are needed to assess the specific associations of smoking in the preconception and childhood periods with offspring outcomes.´*

**Comment 7**

Please report your abstract according to PRISMA for abstracts, following the PLOS Medicine abstract structure (Background, Methods and Findings, Conclusions) <https://eur01.safelinks.protection.outlook.com/?url=http%3A%2F%2Fwww.plosmedicine.org%2Farticle%2Finfo%3Adoi%2F10.1371%2Fjournal.pmed.1001419&amp;data=02%7C01%7Cv.jaddoe%40erasmusmc.nl%7C244ad0d7cfef4b65e27b08d7bea83d18%7C526638ba6af34b0fa532a1a511f4ac80%7C0%7C0%7C637187502100435363&amp;sdata=IHz26%2B4yZgz5lCLrvSPam2k0Q481lzknC0%2BegnIsEd4%3D&amp;reserved=0>

***Response:*** *We have performed the appropriate changes in the abstract according to PRISMA and PLOS Medicine guidelines.*

**Comment 8**

Abstract Background: Please expand upon the context of why the study is important. The final sentence should clearly state the study question.

***Response:*** *We have rewritten the* ***Abstract Background*** *as follows (Page 4, Lines 2-7): ´****Background:*** *Fetal smoke exposure is a common and key avoidable risk factor for birth complications and seems to influence later risk of overweight. It is not clear whether this increased risk is also present if mothers smoke during the first trimester only or reduce the number of cigarettes during pregnancy, or when only fathers smoke. We aimed to assess the associations of parental smoking during pregnancy, specifically of quitting or reducing smoking and maternal and paternal smoking combined, with preterm birth, small size for gestational age (SGA) and childhood overweight.´*

**Comment 9**

Please combine the Methods and Findings components of your Abstract under one subheading of ‘Methods and Findings’.

Please include brief demographic details of the populations included in the meta-analysis (e.g. age ranges, nationalities, parity of women, etc.), years during which the studies took place, and further details of the study settings (i.e. which countries in Europe and North America; from where were women recruited e.g. hospitals, community settings, etc.).

It is not clear how many studies include parental smoking and how many are just maternal or paternal - please mention this in the abstract.

Please include the important dependent variables that are adjusted for in the analyses.

There is no need to define OR and CI in the abstract as these are standard abbreviations.

In the last sentence of the Abstract Methods and Findings section, please describe the main limitation(s) of the study's methodology.

Lines 41-44 - Please quantify the results presented with OR and 95% CI

***Response:*** *We have performed the corresponding changes and the* ***Abstract, Methods and Findings*** *reads now as follows (Pages 4 and 5, Lines 8-28): ´****Methods and Findings:*** *We performed an individual participant data meta-analysis among* *229,158 families from 28 pregnancy/birth cohorts from Europe and North America. All 28 cohorts had information on maternal smoking, and 16 had also information on paternal smoking. In total, 22 cohorts were population-based with birth years ranging from 1991 to 2015. The mothers median age was 30.0 years and most mothers were medium or highly educated. We used multilevel binary logistic regression models adjusted for maternal and paternal sociodemographic and lifestyle related characteristics. Compared with non-smoking mothers, maternal first trimester smoking only was not associated with adverse birth outcomes but was associated with a higher risk of childhood overweight (OR 1.17 (95% CI 1.02-1.35)). Children from mothers who continued smoking during pregnancy had higher risks of preterm birth (OR 1.08 (95% CI 1.02-1.15)), small size for gestational age (OR 2.15 (95% CI 2.07-2.23)) and childhood overweight (OR 1.42 (95% CI 1.35-1.48)). Mothers who reduced the number of cigarettes between the first and third trimester, without quitting, still had a higher risk of small size for gestational age, but the effect estimate was smaller (OR 1.89 (95% CI 1.52-2.34) when reducing from 5-9 to ≤4 cigarettes/day; OR 2.79 (95% CI 2.39-3.25) and OR 1.93 (95% CI 1.46-2.57) when reducing from ≥10 to 5-9 and ≤ 4 cigarettes/day, respectively). Reducing the number of cigarettes during pregnancy did not affect the risks of preterm birth and childhood overweight. Among non-smoking mothers, paternal smoking was associated with childhood overweight (OR 1.21 (95% CI 1.16-1.27)), but not with adverse birth outcomes. Limitations of this study include the self-report of parental smoking information and the possibility of residual confounding. Also, as this study only included participants from Europe and North America, results need to be interpreted with caution with respect to other populations.´*

**Comment 10**

Please replace ‘Interpretation’ with the ‘Conclusions’. Please begin this section with "In this study, we observed ..." or similar. Please address the study implications, emphasizing what is new without overstating your conclusions.

***Response:*** *We have rewritten the* ***Abstract,*** ***Conclusions Section*** *(Page 5, Lines 29-34): ´****Conclusions:*** *In this study, we observed that as compared to non-smoking during pregnancy, quitting smoking in the first trimester is associated with the same risk of preterm birth and small size for gestational age, but with a higher risk of childhood overweight. Reducing the number of cigarettes, without quitting, has limited beneficial effects. Paternal smoking seems to be associated, independently of maternal smoking, with the risk of childhood overweight. Population strategies should focus on maternal and paternal smoking prevention before or at the start, rather than, during pregnancy.´*

**Comment 11**

Introduction

Please expand your Introduction to outline past research and explain the need for and potential importance of your study. Indicate whether your study is novel and how you determined that. If there has been a systematic review of the evidence related to your study (or you have conducted one), please refer to and reference that review and indicate whether it supports the need for your study.

Line 59 - please provide the range for % women who smoke during pregnancy, or revise the term ‘range’

Line 61 - Please revise or remove sentence "Also, since paternal and maternal smoking often cluster within families, insight into the effects of combined parental smoking may help to improve family-focused prevention strategies", as prevention strategies are not included in the scope of this study.

***Response:*** *We have revised the* ***Introduction Section*** *as follows (Page 8, Lines 103-126):*

*´****INTRODUCTION***

*One in five women of reproductive age are expected to be tobacco users by 2025 [1]. Although strategies to prevent smoking are globally implemented, up to 25% of women in Western countries smoke during pregnancy [2]. This is a major public health concern, particularly since smoking during pregnancy does not only affect women’s own health, but is also associated with adverse birth and offspring outcomes, such as preterm birth, low birth weight and childhood overweight [3-13]. Preterm birth and low birth weight are a major cause of perinatal morbidity and mortality and childhood overweight is related to a higher risk of cardiovascular disease, premature death and disability in adulthood [14-16].*

*A vast amount of studies observed consistent associations of continued maternal smoking during pregnancy with increased risks of preterm birth, low birth weight and childhood overweight [7,10,11]. However, evidence on critical windows of vulnerability to maternal smoking and changes in smoking behavior during pregnancy remains inconclusive, potentially reflecting between-study heterogeneity of outcome measures and small study sample sizes. Previous studies focused on maternal smoking in first trimester of pregnancy only, consistently showed no associations with preterm birth, but showed conflicting results for the risks of low birth weight and childhood overweight [8,9,17-21]. Also, the associations of paternal smoking during pregnancy with preterm birth, low birth weight and childhood overweight have been scarcely studied and remain unclear [20,22,23]. To our knowledge, no large sample size studies assessed the associations of maternal smoking during first trimester only, of reducing the number of cigarettes during pregnancy, or of paternal smoking only with birth and childhood outcomes.*

*We conducted an individual participant data meta-analysis among 229,158 singleton births from 28 pregnancy and birth cohort studies in Europe and North America to assess the associations of parental smoking during pregnancy with preterm birth, small size for gestational age (SGA) and childhood overweight. We were specifically interested in the associations of quitting or reducing smoking during pregnancy, and of combined maternal and paternal smoking patterns with birth and offspring outcomes.´*

**Comment 12**

Methods

Please incorporate eFigure 1 into the main text of your manuscript.

Please add the following statement, or similar, to the Methods: "This study is reported as per the Preferred Reporting Items for Systematic Reviews and Meta-Analyses (PRISMA) guideline (S1 Checklist)."

When completing the PRISMA checklist, please use section and paragraph numbers, rather than page numbers.

Please complete your PRISMA checklist - several boxes not ticked. We note that some form of search strategy was used (even if not like a conventional meta-analysis) - please elaborate on how cohorts were identified (line 75 - "cohort studies were invited") and how eligibility for inclusion in the meta-analysis was decided?

Please expand upon the methods used to check quality and heterogeneity, which are paramount for meta-analyses.

We note that all covariates are categorised. Please ensure that certain variables such as age not categorised (please refer to report from Reviewer 1)

Please confirm that written or oral informed consent was obtained in all cohort studies included.

***Response:*** *We have moved the flow chart into the main manuscript and are now presenting as Figure 1. We have completed the PRISMA checklist as requested. We have performed the following textual changes to clarify our strategy for selection and inclusion of cohorts:*

***´Inclusion criteria and participating cohorts***

*This study was part of an international LifeCycle Project (*[*https://lifecycle-project.eu*](https://lifecycle-project.eu)*) collaboration on maternal obesity and childhood outcomes [24-28]. Pregnancy and birth cohort studies were eligible for inclusion if they included mothers with singleton live-born children who were born from 1989 onwards, had information available on maternal pre/early-pregnancy body mass index (BMI) and at least one offspring measurement (birth weight or childhood BMI). We identified eligible cohorts from existing collaborations on childhood health (EarlyNutrition Project, CHICOS Project, www.birthcohorts.net assessed until July 2014). Fifty cohorts from Europe, North America and Oceania were identified and invited, of which 39 cohorts agreed to participate. The cohorts included were approved by their local institutional review boards and written informed consent from all participants or parents was obtained. Eleven cohorts were excluded from the current analysis due to no information on maternal smoking patterns or only non-smoking mothers in their cohort. In total, 28 cohorts comprising data on 229,158 singleton births were included (****Fig 1****). Twenty-two of the 28 cohorts defined themselves as regionally or nationally-based studies, four as hospital-based (Co.N.ER, EDEN, GASPII, LUKAS), one as internet users-based (NINFEA), and one as studying selected populations (FCOU). All cohorts provided written consent for using their data. Anonymized datasets were stored on a single central secured data server with access for the main analysts (EP, SS) only. This study is reported according to the Preferred Reporting Items for Systematic Reviews and Meta-Analyses (PRISMA) guideline (S1 Checklist).´ (Page 9, Lines 130-147)*

*We have clarified the methods used to check heterogeneity in the following sentence in the* ***Methods****,* ***Statistical analysis*** ***Section*** *(Page 12, Lines 208-211):* **´***Also, we conducted 2-stage random effects meta-analyses for the core associations and tested for heterogeneity between the cohorts estimates with the I^2^ test [33,35]. To express the uncertainty associated with I^2^ estimates, we calculated the corresponding 95% confidence intervals (CI) [36].´*

*We have added to the* ***Discussion Section*** *(Page 26, Lines 476-479): ´Since we used original, individual participant data, we did not formally assess the quality of the individual studies included. We are aware that our study cannot overcome potential limitations of individual studies in terms of their design and conduct, differences in the definitions of exposure and outcome data, and variation in missing data.´*

*Due to the strategy used to handle missing data, the confounding factors were included in the models as categorical covariates. In order to understand the influence on our results of using maternal age and BMI as categorical covariates, we have performed complete cases analysis using maternal age and BMI categorically or continuously. We observed similar effect estimates when using categorical or continuous maternal BMI and age. We have performed the following textual changes:*

***Covariates*** *(Page 11, Lines 182-191)*

*Information on covariates was mostly assessed using questionnaires. Most covariates were provided by cohorts as categorical variables: child’s sex, maternal educational level (low, medium, high), parity (nulliparous, multiparous), and alcohol consumption during pregnancy (yes, no). To allow handling of missing data, continuous covariates were categorized: maternal age (defined on the basis of data availability: ˂25.0 years, 25.0-29.9 years, 30.0-34.9 years, and ≥35.0 years) and pre- or early pregnancy maternal and paternal BMI (underweight [<18.5 kg/m^2^], normal weight [18.5–24.9 kg/m^2^], overweight*

*[25.0–29.9 kg/m^2^], and obesity [≥30.0 kg/m^2^]). Maternal ethnicity was not included since most cohorts were largely Caucasian and there was a high percentage of missings. Covariates per cohort are described in* ***S2 Table****.*

**´Statistical analysis** *(Page 12, Lines 211-220)*

*All covariates were categorized and missing values were added as an additional group to prevent exclusion of non-complete cases. If information on a covariate was available for less than 50% of the cohort sample used for each analysis, available information was not used and the corresponding data for that full cohort sample was assigned to the missing category.* *We conducted a sensitivity analysis with complete cases only. Also, to explore the influence on our results of using maternal age and BMI as categorical covariates, we repeated the complete cases analysis using these covariates continuously. The statistical analyses were performed using the Statistical Package of Social Sciences version 24.0 for Windows (SPSS Inc, Chicago, IL, USA) and Review Manager (RevMan) version 5.3 of the Cochrane Collaboration (The Nordic Cochrane Centre, Copenhagen, Denmark).´*

***Discussion*** *(Page 27, Lines 501-507)*

***´****Missing values of covariates were used as an additional group. This approach has been commonly used in large IPD meta-analyses due to the constraints in applying more advanced imputation strategies. Although we cannot disregard the possibility of bias, we consider it unlikely considering the relatively small percentage of missings [57]. We observed similar results when we conducted a complete case analysis (S7 Table). Also, similar associations were observed when adjusting for maternal age and BMI as categorical or continuous covariates (S7 and S8 Tables).´*

**Comment 13**

Results

Lines 161-7 - Please quantify the results presented with OR and 95% CI

All tables - please define all abbreviations used in the table legend (except cohort names).

Please incorporate supplementary figures into the main text.

***Response:*** *We have added the corresponding OR and 95% CI in the following sentences (Page 18, Lines 310-319):* ***´Table 3*** *shows that, compared with mothers who did not smoke during pregnancy, mothers who quitted smoking from first-to-third trimester had similar risks of delivering SGA infants. Reducing the number of cigarettes, without quitting, from first-to-third trimester lowered the risks of delivering SGA infants, but risks were still higher compared with non-smoking mothers (OR 1.89 (1.52-2.34) when reducing from 5-9 to ≤4 cigarettes/day; 2.79 (2.39-3.25) and 1.93 (1.46-2.57) when reducing from ≥10 to 5-9 and ≤ 4 cigarettes/day, respectively). Mothers who increased the number of cigarettes from first-to-third trimester increased their risks of delivering SGA infants (OR 2.43 (2.05-2.89) and 2.47 (1.71-3.58) when increasing from ≤4 to 5-9 and ≥10 cigarettes/day, respectively; and 2.70 (2.35-3.10) when increasing from 5-9 to ≥10 cigarettes/day). Changes in maternal smoking from first-to-third trimester did not influence the risks of preterm birth and childhood overweight.´*

We have defined all abbreviations used in the table legend and have moved supplementary figures into the main text.

**Comment 14**

Discussion

Please remove all subheadings throughout your discussion.

Please present and organize the Discussion as follows: a short, clear summary of the article's findings; what the study adds to existing research and where and why the results may differ from previous research; strengths and limitations of the study; implications and next steps for research, clinical practice, and/or public policy; one-paragraph conclusion.

***Response:*** *We have organized the discussion as suggested (Pages 22-28). We have also added one paragraph discussing the implications and next steps for research, clinical practice, and/or public policy as follows (Pages 27-28, Lines 510-526): ´Our results suggest that as compared to mothers who continued smoking throughout pregnancy, mothers who quit smoking during the first trimester have a reduced risk of birth complications. Reducing the number of cigarettes without quitting during pregnancy is still associated with an increased risk of birth complications. The observed effect estimates were small to moderate but are important from a public health perspective since smoking is a common adverse exposure and preterm birth and SGA are among the most frequent birth complications. Also, preterm birth, SGA and childhood obesity are related with adverse health consequences later in life. Our findings suggest that is of great importance to invest in prevention of smoking in women of reproductive age before or at the start of pregnancy. Pregnant women should still be motivated to reduce smoking, even later in pregnancy. The current guidelines focus only on quitting smoking and not reducing, while this can be discouraging for women who find it difficult to quit smoking. These women should be provided with sufficient information about the risks of continued smoking but also about the benefits of reducing their number of cigarettes. Future research should investigate whether quitting smoking in the first trimester or reducing the number of cigarettes during pregnancy is also beneficial for other adverse birth and offspring outcomes. Although we cannot exclude a role of residual confounding and shared family-based characteristics in the associations of paternal smoking with childhood overweight, we recommend that fathers are more closely involved in preconception and pregnancy consultations focused on smoking reduction.´*

**Comment 15**

We note that table 1 quotes numbers for smoking in “early pregnancy only” (72 participants in the BAMSE cohort, for example); eTable 3 has numbers on first trimester maternal smoking (e.g., 483 participants in the BAMSE cohort). Please let us know how these numbers should be interpreted—for example, it may be a simple matter of explaining how “early pregnancy” is defined, as this does not seem to equate to first trimester.

***Response:*** *We have incorrectly used ´Early pregnancy only´ in Table 1, when it should be ´First trimester only´. We have corrected this in Table 1. The numbers in Table 1 and S3 Table are different because Table 1 concerns maternal smoking during first trimester only while S3 Table concerns all smoking in first trimester no matter if mothers stop or continue smoking after first trimester. We have clarified this in the footnotes of the corresponding tables.*

**Academic editor**

**Comment 1**

The authors imply causation in several places - eg "Quitting smoking in the first trimester place mothers at the same risk of preterm birth and small size for gestational age as non-smoking mothers."

I think the language should be tempered throughout to make it clear that the findings are based on observational data and associations can be seen, but causation cannot be assessed. The authors do discuss, that "shared family-based lifestyle and genetic characteristics are potential underlying mechanisms" for childhood overweight; and this makes sense. However, the abstract implies that maternal and paternal smoking are directly linked to childhood overweight.

***Response:*** *We agree and have made the changes accordingly.* *Please see also our response to* ***Comment 1*** *of the* ***Editors.***

**Reviewer #1**

This paper describes an individual participant data meta-analysis (IPD) of the relationship between paternal smoking and child outcomes. I was asked for a statistical report and I interpret that to include all aspects of the design and conduct of the study.

***Response:*** *We thank the reviewer for the comments. Please find our responses to specific comments below.*

**Comment 1**

Page 5 Just for the record I think using 230000 in the title is fine even though strictly speaking there are only 229158. If anyone objects I would replace it with 220000+ rather than the exact number.

***Response:*** *We agree.* *Please see our response to* ***Comment 4*** *of the* ***Editors.***

**Comment 2**

Page 7 The authors have not claimed that this is a systematic review intending to cover all known studies. However the process of selection is not clear, at least to me, and we could use a bit more detail.

***Response:*** *We have clarified the* ***Methods Section.*** *Please see our response to* ***Comment 12*** *of the* ***Editors.***

**Comment 3**

Page 7 The countries represented are all in Europe apart from one study from the United States. Apart from Ukraine they all seem to be what the World Bank calls high-income. Is this an artefact of the selection method presented here? Does the focus primarily on high income countries affect generalisability?

***Response:*** *We have addressed this in the* ***Discussion Section*** *(Page 26, Lines 466-473): ´We performed an individual participant data meta-analysis of prospective cohort studies to investigate the associations of parental smoking during pregnancy with preterm birth, SGA and childhood overweight.* *We*

*included data from cohort studies in Europe and North America, so our findings are mainly applicable to populations in developed countries. Inclusion of data from other regions could have led to differences in prevalence of maternal and paternal smoking, birth complications, childhood overweight and ethnic and sociodemographic characteristics, complicating or limiting the possibility to do a meta-analysis. Further studies are needed to assess these associations among populations from low-income and middle-income countries.****´***

**Comment 4**

Page 7 and 8 Most of the variables have been categorised. As I am sure the authors know categorising an essentially continuous variable wastes information (Altman and Royston, 2006; Royston et al., 2006) and leads to models which are often implausible as they predict the effect remaining at within categories and then jumping to a new value at the category boundary. I can see why in an IPD study this is sometimes inevitable as primary studies used incompatible classifications (in which case perhaps say so) but why categorise the anthropometric measures which presumable were recorded in grams and centimetres everywhere.

***Response:*** *Please see also our response to* ***Comment 12*** *of the* ***Editors.*** *We have repeated the analyses using the* *continuous outcomes, i.e., the gestational age at birth, sex- and gestational-age adjusted birth weight SDS and childhood sex- and age-adjusted BMI SDS.* *We have performed the following textual changes:*

***Statistical analysis*** *(Page 12, Lines 206-208):*

*´As sensitivity analyses, we repeated all models for gestational age at birth, sex- and gestational-age adjusted birth weight SDS and childhood sex- and age-adjusted BMI SDS.´*

**Results***:*

*´We observed similar results when we used the continuous outcomes, except for the association of first trimester only smoking with childhood BMI SDS, which was in the same direction but no longer significant (S4 Table).´ (Page 15, Lines 248-250)*

*´Similar results were observed when assessing the associations of the changes in maternal smoking during pregnancy with the continuous outcomes (S5 Table).´ (Page 18, Lines 319-321)*

*´Similar results were observed for the combined maternal and paternal smoking with the continuous outcomes (S6 Table).´ (Page 21, Lines 342-343)*

**Comment 5**

Page 10 Confidence intervals for I2 would also be helpful to reveal our uncertainty about the heterogeneity. I know we did not use to use them but I am persuaded by Ioannidis et al. (2007) that we should. I am rather surprised by the low levels of heterogeneity seen in the supplementary figures. These are not gigantic samples but they are all fairly substantial and following the arguments in Rucker et al. (2008) I would have expected more heterogeneity.

***Response:*** *We have calculated the confidence intervals for I^2^ and are now presenting in footnotes of the corresponding figures. We have performed the following textual changes:*

***Methods, Statistical analysis Section*** *(Page 12, Lines 208-211):*

*Also, we conducted 2-stage random effects meta-analyses for the core associations and tested for heterogeneity between the cohorts estimates with the I^2^ test [33,35]. To express the uncertainty associated with I^2^ estimates, we calculated the corresponding 95% confidence intervals (CI) [36].*

***Results*** *(Page 15, Lines 250-259)*

*´We observed similar results when using 2-stage random effects models (****Fig 2, 3 and 4****). We observed low-to-moderate heterogeneity between the cohorts estimates (I^2^ estimates range from 0 to 47%; corresponding CI are presented in the footnotes of Fig 2, 3 and 4). Only the cohort-specific results for the associations of maternal continued smoking with SGA showed high heterogeneity between estimates (I^2^ 75% ( 95% CI 56% - 86%)). Almost all cohorts were included in the analyses for continued smoking while only roughly half had information on first trimester only smoking. When restricting the 2-stage continued smoking models to the cohorts also with information on first trimester only smoking, we observed a lower heterogeneity between estimates (I^2^ 23% (95% CI 0% - 65%)) but the pooled effect estimate remained similar (S1 Fig).´*

***Discussion*** *(Page 27, Lines 495-501)*

*´Overall, we observed low-to-moderate heterogeneity in the 2-stage random effects models, which might be due to the inclusion of mostly high-income and Caucasian cohorts. However, we observed high heterogeneity between the cohorts for the associations of maternal continued smoking with SGA. This might be in part explained by differences in pattern and dosage of maternal and paternal smoking between cohorts. When we restricted the 2-stage continued smoking models to the cohorts that also had information on first trimester only smoking, we observed a substantially lower heterogeneity between estimates.´*

**Comment 6**

Page 10 and Table 3 I think the smaller numbers in Table 3 might justify more caution in interpreting any which fail to reach some level of statistical significance.

***Response:*** *We have acknowledged this limitation in the* ***Discussion Section*** *(Page 26, Lines 473-476):* ***´****The large sample size enabled us to investigate the effects of changing smoking habits and paternal smoking. However, we might have been underpowered to detect associations in the analyses looking at maternal only first trimester smoking and the change in smoking habits from first to third trimester, due to small sample sizes.****´***

**Comment 7**

Page 10 The interactions presented here have been given as estimates for the separate strata and a p-value for the test but we do not have an estimate for the difference or interaction. If the authors have tested for interactions formally then why not present the relative odds ratio so that we can see how much one is greater than the other with a confidence interval?

***Response:*** *We have not formally tested for interaction in these analyses. We created a single variable combining the maternal and paternal smoking information in different categories and have explored the associations for each category using the maternal and paternal non-smoking as reference category. Additionally, we have investigated if the effect estimates between the categories statistically differed. We have clarified this information by performing the following textual changes:*

***Parental tobacco smoking*** *(Page 10, Lines 158-163)*

*´To explore the combined effects of maternal and paternal smoking, we combined the maternal and paternal smoking information into six categories: maternal and paternal non-smoking (used as reference category); maternal non-smoking and paternal smoking; maternal first trimester only smoking and paternal non-smoking; maternal first trimester only smoking and paternal smoking; maternal continued smoking and paternal non-smoking; and maternal continued smoking and paternal smoking.´*

***Statistical analysis*** *(Page 11, Lines 201-203)*

***´****Finally, we used similar models to investigate the combined associations of both maternal and paternal smoking with the risks of these outcomes. We assessed whether the effect estimates between categories statistically differed using the formula* $Z=\frac{\beta1- \beta2}{\sqrt{{(SE\beta1)}^{2}+{(SE\beta2)}^{2}}}$ *[34].****´***

***Results*** *(Page 21, Lines 333-342)*

*Among mothers who did not smoke during pregnancy, paternal smoking tended to be associated with higher risks of preterm birth (OR 1.06 (1.00-1.12)), SGA (OR 1.04 (1.00-1.09)) and childhood overweight (OR 1.21 (1.16-1.27)) (****Table 4****). Among mothers who smoked during first trimester only, paternal smoking was not associated with preterm birth or SGA, but was associated with a higher risk of childhood overweight (OR 1.36 (1.02-1.80)). Among mothers who continued smoking during pregnancy, paternal smoking further increased the risks of SGA and childhood overweight (both Z-score P-value for differences in effect sizes between categories­ <0.0001), but not the risk of preterm birth. Children whose mothers continued smoking during pregnancy and whose fathers also smoked had the highest risks of being born preterm (OR 1.10 (1.02-1.19)), and SGA (OR 2.37 (2.26-2.49)), and of childhood overweight (OR 1.76 (1.65-1.87)).*

**Comment 8**

Page 32 Table 1 Of the three outcomes this shows us the prevalence of childhood overweight but not the prevalence of pre-term birth or of small for gestational age. Since they are what is being analysed that would seem helpful information to add.

***Response:*** *We have added the cohort specific and total group prevalence of preterm birth and small for gestational age at birth to* ***Table 1.*** *We have rewritten the following sentence in* ***the Results Section*** *(Page 12, Lines 227-228): ´In total, 4.7% of children were born preterm, 10.0% were small for gestational age at birth and 20% were in the overweight group.´*

**Comment 9**

Page 32 Table 1 In the footnote here what exactly does `selected samples' mean? Are these clinical samples with restricted ranges on some variables?

***Response:*** *We have clarified this in the footnote of* ***Table 1****: ´NA, not available (not collected or not provided) or not applicable (gestational age at birth (FCOU, GINIplus, LISAplus, LUKAS) and birth weight (GINIplus, LISAplus) due to study samples restricted to specific ranges of gestational age and weight at birth)´.*

**Comment 10**

The 2-stage estimates presented in the supplement are useful because they give estimates of heterogeneity and also the important information that not all studies contribute to all estimates. Unless I have missed this it seems important information to be included in the main report since the sample on which first trimester only versus none is being compared is not the same sample as the continued versus none. The plots are fine in the supplement but they are under-played in the main text I think.

***Response:*** *We have moved the plots to the main text as requested by* ***Editors*** *in* ***Comment 13.*** *We have more extensively described the 2-stage analyses and results. For textual changes, please see our response to* ***Comment 5*** *of* ***Reviewer#1****.*

**Reviewer #2**

This study stems from the knowledge that prenatal exposure to maternal smoking carries risks for the baby and it investigates the important question of whether quitting smoking during early pregnancy reduces the risks of birth complications and childhood obesity, compared to continued smoking. The authors have conducted a powerful study by meta-analysing data from 28 European cohorts reaching an impressive N=229158 families. They conclude that quitting early in pregnancy is beneficial in reducing the risk of low birth weight. This study is commendable since it is powerful and addresses an important and needed question with immediate clinical relevance. However, before its publication I would like the authors to carry out revisions according to the following points.

***Response:*** *We thank the reviewer for the comments. Please find our responses to specific comments below.*

**Comment 1**

The authors mention in the Introduction that smoking leads to, amongst other outcomes, congenital abnormalities, still birth and sudden infant death syndrome, which are major complications, but the study focuses on pre-term birth, low birth weight and childhood overweight, potentially less life-threatening. What was the reason to leave out the most detrimental outcomes from this study? If this study is to be translated to a clinical application it would need to be relevant to families in reducing risks of major complications. Perhaps the authors could emphasised how detrimental pre-term births, low birth weight and being overweight in childhood are for long-term health to add context to this research.

***Response:*** *We designed this study with a specific interest in preterm births, low birth weight and being overweight, but agree that the other outcomes are also of interest. We have emphasized how detrimental preterm births, low birth weight and being overweight in childhood are for long-term health in the* ***Introduction Section****. Please see our response to* ***Comment 11*** *of the* ***Editors.*** *We have also* *highlighted the need for future research to look at these associations in relation to other adverse major complications in the* ***Discussion Section*** *(Page 28, Lines 521-523): ´Future research should investigate whether quitting smoking in the first trimester or reducing the number of cigarettes during pregnancy is also beneficial for other adverse birth and offspring outcomes.´*

**Comment 2**

The Methods (either main text or supplemental) section needs an explanation per cohort on how smoking and the covariates were measured and derived. It is very unlikely that the type of information is the same across all these numerous studies. For instance, was the question asked to the mothers whether they smoked in the first trimester and similarly for the later time-points or was the question whether they smoked in the last two weeks or currently relative to a questionnaire? Some studies contain many variables related to smoking, so it is important to know what was used to create the smoking variables used here. Even if questionnaires are aimed at first trimester, depending on the study they might have been returned at various times during pregnancy. Some women might have been enrolled late in the study etc. so the accuracy of the time of this self-reported information in relation to the time in pregnancy can vary and it is important to specify all this information to understand the results and to be able to reproduce the study. Particularly as some cohorts are much bigger in size than others and they might have affected the results more.

***Response:*** *We have acknowledged this as limitation in the* ***Discussion Section*** *(Page 26, Lines 480-483): ´Parental smoking information during pregnancy was self-reported. For active smoking, correlations between cotinine measurements and self-reported smoking habits are high [54]. We have no information on the specific question asked or the timing in which it was asked, which might have differed across cohorts and influenced our results.´*

**Comment 3**

At lines 136-137: What was the reason for categorising the missing participants as an additional group rather than conducting multiple imputation? This approach could add bias in the estimates as shown for instance by Groenfeld et al (CMAJ. 2012 Aug 7; 184(11): 1265-1269)?

***Response:*** *Due to data availability and the size of the dataset, we were not able to apply more advanced imputation strategies. We used a missing value category to deal with missing data for covariates. As sensitivity analyses, we have now performed complete case analyses. For further details and textual changes, please see our response to* ***Comment 12*** *of the* ***Editors.***

**Comment 4**

The authors have not commented on the limitation that for some of the analyses the sample size was very small (for instance in the dose-stratified analyses in Table 3) and when they compare the effect of a change in habit between first-trimester-only smoking to continued or the effect of a dose change they are effectively comparing the different associations (odds ratios). However, differences in samples sizes lead to differences in power and therefore the associations are not always comparable. They should make conclusions only regarding the associations for which there is enough evidence to conclude that there is a risk, for instance the effect of continued smoking (based on N~5000) on small for gestational age. They cannot rule out an effect of smoking only in the first trimester (based on N~200).

***Response:*** *We agree. Please see our response to* ***Comment 6*** *of the* ***Reviewer#1.***

**Comment 5**

Some of the effects considered have confidence interval very close to 1, so the authors should be more cautious in concluding a risk since even if there was an effect it might be small and not necessarily meaningful.

***Response:*** *We have addressed this in the* ***Discussion Section*** *(Pages 27 and 28, Lines 513-516): ´The observed effect estimates were small to moderate but are important from a public health perspective since smoking is a common adverse exposure and preterm birth and SGA are among the most frequent birth complications. Also, preterm birth, SGA and childhood obesity are related with adverse health consequences later in life.´*

**Comment 6**

Could the effect of paternal smoking on 'childhood overweight' suggest that there is some residual confounding?

***Response:*** *We agree with the reviewer that the association of paternal smoking with childhood overweight might suggest residual confounding. We have discussed this in the* ***Discussion Section*** *(Page 24, Lines 431-436): ´In the current study, paternal smoking among non-smoking mothers was associated with a higher risk of childhood overweight and tended to be associated with higher risks of preterm birth and SGA. This suggests that paternal smoking may be, independently of maternal smoking, associated with childhood overweight. However, we cannot exclude the possibility of residual confounding by factors not or insufficiently measured in the studies.´*

**Comment 7**

The authors should state clearly in the abstract, discussion and conclusions that this is an observational study, rather than causal, and the difference in associations between different smoking behaviours might be due to familial characteristics rather than different exposure to smoke, which could be measured by cotinine levels for instance, at different timepoints in pregnancy in relation to birth outcomes.

***Response:*** *We have addressed this in the* ***Abstract and Discussion Sections*** *as follows:*

***Abstract*** ***Section*** *(Page 4, Lines 25-26)*

*´Limitations of this study include the self-report of parental smoking information and the possibility of residual confounding.´*

**Discussion Section** *(Page 27, Lines 507-509)*

*´Although we adjusted for multiple lifestyle-related factors, we cannot exclude residual confounding by other environmental lifestyle-related factors. From the current observational data, no conclusions can be drawn on the causality of the observed associations.´*

**Comment 8**

Could the authors investigate more the issue of high heterogeneity in the small for gestational age analysis by conducting some sensitivity analysis? For instance they could conduct the analysis only including the cohorts that have both data (first trimester and continued) and compare the associations found across these to check that they remain similar to what they have found. The authors should consider the effect that excluding large cohorts from the first trimester data could have on the continued smoking analyses.

***Response:*** *Please see our response to* ***Comment 5*** *of* ***Reviewer#1.***

**Comment 9**

At lines 104-105: what category was used as reference for gestational age? The authors mentioned the definition used for SGA and that "appropriate and large size for gestational age were used as the reference group". Do they mean that everyone else not in the category for SGA was the reference group?

***Response:*** *We have rewritten the following sentences (Page 10, Lines 167-173): ´Preterm birth was defined as <37 weeks of gestation, and full-term birth (≥37 weeks) was used as the reference group in the analyses [29]. We created sex- and gestational age-adjusted birth weight standard deviation scores (SDS) based on a North European reference chart [30]. SGA at birth was defined per cohort as sex- and gestational age-adjusted birth weight below the 10^th^ percentile. The reference group used in the analyses comprises children born appropriate and large size for gestational age (i.e., cohort-specific sex- and gestational age-adjusted birth weight above the 10^th^ percentile).´*

**Comment 10**

At lines 159-160: Could the authors add info on heterogeneity in the other models too? Could they also add in the Discussion what could have contributed to the high heterogeneity for the small for gestational age outcome?

***Response:*** *Please see our response to* ***Comment 5*** *of* ***Reviewer#1.***

**Reviewer #3**

This is an individual participant data meta-analysis of prospective cohort studies to examine the associations of prenatal parental smoking with adverse birth outcomes and childhood overweight. It is a well-described manuscript, with large study sample. It would be much better if the authors more discuss the biological mechanisms in the discussion session. For example, the authors referred to cadmium and DNA methylation, which is very interesting, but do they explain the different results in the associations of the outcomes?

***Response:*** *We have more extensively discussed the underlying mechanisms of the observed associations in the* ***Discussion Section*** *(Pages 25-26, Lines 451-463): ´Various components of tobacco smoke might be involved in the mechanistic pathway towards adverse birth outcomes and childhood overweight. Both nicotine and carbon monoxide are reported to reduce placental blood flow [47]. Nicotine stimulates acetylcholine receptors, which release a multitude of vasoactive catecholamines and peptides, which in turn reduce blood flow through vasoconstriction [47]. Carbon monoxide competes with oxygen for binding sites on the transport protein hemoglobin causing hypoxia [48]. Chronic hypoxia interferes with the maternal circulatory adjustments to pregnancy which can be another cause of reduced placental blood flow [49]. Uterine blood flow is essential for uterine, placental and fetal growth. Several mechanisms for nicotine-induced alterations in overweight risks have been proposed, including stimulation of the fetal hypothalamic-pituitary axis [50]. It has been suggested that cadmium, present in tobacco smoke, modulates oxytocin receptor function, proposing a role in the pathophysiology of preterm birth [48]. Recent studies have found an association between maternal smoking during pregnancy and birth weight with a mediating role of DNA methylation [51-53]. Further research is needed to assess such possible mechanisms.´*

**REFERENCES**

1.	Samet JM, Yoon SY. Women and the tobacco epidemic: challenges for the 21st century. The World Health Organization, in collaboration with the Institute for Global Tobacco Control, Johns Hopkins School of Public Health; 2001.

2.	Lange S, Probst C, Rehm J, Popova S. National, regional, and global prevalence of smoking during pregnancy in the general population: a systematic review and meta-analysis. Lancet Glob Health. 2018;6: e769-e776.

3.	Cnattingius S. The epidemiology of smoking during pregnancy: smoking prevalence, maternal characteristics, and pregnancy outcomes. Nicotine Tob Res. 2004;6 Suppl 2: S125-140.

4.	Hackshaw A, Rodeck C, Boniface S. Maternal smoking in pregnancy and birth defects: a systematic review based on 173 687 malformed cases and 11.7 million controls. Hum Reprod Update. 2011;17: 589-604.

5.	Marufu TC, Ahankari A, Coleman T, Lewis S. Maternal smoking and the risk of still birth: systematic review and meta-analysis. BMC Public Health. 2015;15: 239.

6.	Zhang K, Wang X. Maternal smoking and increased risk of sudden infant death syndrome: a meta-analysis. Leg Med (Tokyo). 2013;15: 115-121.

7.	Shah NR, Bracken MB. A systematic review and meta-analysis of prospective studies on the association between maternal cigarette smoking and preterm delivery. Am J Obstet Gynecol. 2000;182: 465-472.

8.	Raisanen S, Sankilampi U, Gissler M, Kramer MR, Hakulinen-Viitanen T, Saari J, et al. Smoking cessation in the first trimester reduces most obstetric risks, but not the risks of major congenital anomalies and admission to neonatal care: a population-based cohort study of 1,164,953 singleton pregnancies in Finland. J Epidemiol Community Health. 2014;68: 159-164.

9.	Moore E, Blatt K, Chen A, Van Hook J, DeFranco EA. Relationship of trimester-specific smoking patterns and risk of preterm birth. Am J Obstet Gynecol. 2016;215: 109.e101-106.

10.	Kramer MS. Determinants of low birth weight: methodological assessment and meta-analysis. Bull World Health Organ. 1987;65: 663-737.

11.	Rayfield S, Plugge E. Systematic review and meta-analysis of the association between maternal smoking in pregnancy and childhood overweight and obesity. J Epidemiol Community Health. 2016;71: 162-173.

12.	Durmus B, Heppe DH, Taal HR, Manniesing R, Raat H, Hofman A, et al. Parental smoking during pregnancy and total and abdominal fat distribution in school-age children: the Generation R Study. Int J Obes (Lond). 2014;38: 966-972.

13.	Albers L, Sobotzki C, Kuss O, Ajslev T, Batista RF, Bettiol H, et al. Maternal smoking during pregnancy and offspring overweight: is there a dose-response relationship? An individual patient data meta-analysis. Int J Obes (Lond). 2018;42: 1249-1264.

14.	Saigal S, Doyle LW. An overview of mortality and sequelae of preterm birth from infancy to adulthood. Lancet. 2008;371: 261-269.

15.	Daniels SR, Jacobson MS, McCrindle BW, Eckel RH, Sanner BM. American Heart Association Childhood Obesity Research Summit Report. Circulation. 2009;119: e489-517.

16.	Ludvigsson JF, Lu D, Hammarstrom L, Cnattingius S, Fang F. Small for gestational age and risk of childhood mortality: A Swedish population study. PLoS Med. 2018;15: e1002717.

17.	Blatt K, Moore E, Chen A, Van Hook J, DeFranco EA. Association of reported trimester-specific smoking cessation with fetal growth restriction. Obstet Gynecol. 2015;125: 1452-1459.

18.	Jaddoe VW, Troe EJ, Hofman A, Mackenbach JP, Moll HA, Steegers EA, et al. Active and passive maternal smoking during pregnancy and the risks of low birthweight and preterm birth: the Generation R Study. Paediatr Perinat Epidemiol. 2008;22: 162-171.

19.	McCowan LM, Dekker GA, Chan E, Stewart A, Chappell LC, Hunter M, et al. Spontaneous preterm birth and small for gestational age infants in women who stop smoking early in pregnancy: prospective cohort study. BMJ. 2009;338: b1081.

20.	Harris HR, Willett WC, Michels KB. Parental smoking during pregnancy and risk of overweight and obesity in the daughter. Int J Obes (Lond). 2013;37: 1356-1363.

21.	Grzeskowiak LE, Hodyl NA, Stark MJ, Morrison JL, Clifton VL. Association of early and late maternal smoking during pregnancy with offspring body mass index at 4 to 5 years of age. J Dev Orig Health Dis. 2015;6: 485-492.

22.	Durmus B, Kruithof CJ, Gillman MH, Willemsen SP, Hofman A, Raat H, et al. Parental smoking during pregnancy, early growth, and risk of obesity in preschool children: the Generation R Study. Am J Clin Nutr. 2011;94: 164-171.

23.	Inoue S, Naruse H, Yorifuji T, Kato T, Murakoshi T, Doi H, et al. Impact of maternal and paternal smoking on birth outcomes. J Public Health (Oxf). 2016;39: 1-10.

24.	LifeCycle Project-Maternal Obesity and Childhood Outcomes Study Group, Voerman E, Santos S, Inskip H, Amiano P, Barros H, et al. Association of Gestational Weight Gain With Adverse Maternal and Infant Outcomes. JAMA. 2019;321: 1702-1715.

25.	Voerman E, Santos S, Patro Golab B, Amiano P, Ballester F, Barros H, et al. Maternal body mass index, gestational weight gain, and the risk of overweight and obesity across childhood: An individual participant data meta-analysis. PLoS Med. 2019;16: e1002744.

26.	Santos S, Voerman E, Amiano P, Barros H, Beilin LJ, Bergstrom A, et al. Impact of maternal body mass index and gestational weight gain on pregnancy complications: an individual participant data meta-analysis of European, North American and Australian cohorts. BJOG. 2019;126: 984-995.

27.	Santos S, Eekhout I, Voerman E, Gaillard R, Barros H, Charles MA, et al. Gestational weight gain charts for different body mass index groups for women in Europe, North America, and Oceania. BMC Med. 2018;16: 201.

28.	Patro Golab B, Santos S, Voerman E, Lawlor DA, Jaddoe VWV, Gaillard R, et al. Influence of maternal obesity on the association between common pregnancy complications and risk of childhood obesity: an individual participant data meta-analysis. Lancet Child Adolesc Health. 2018;2: 812-821.

29.	Tucker J, McGuire W. Epidemiology of preterm birth. BMJ. 2004;329: 675-678.

30.	Niklasson A, Ericson A, Fryer JG, Karlberg J, Lawrence C, Karlberg P. An update of the Swedish reference standards for weight, length and head circumference at birth for given gestational age (1977-1981). Acta Paediatr Scand. 1991;90: 756-762.

34.	Paternoster R, Brame R, Mazerolle P, Piquero A. Using the correct statistical test for equality of regression coefficients Criminology. 1998;36: 859-866.

33.	Debray TP, Moons KG, Abo-Zaid GM, Koffijberg H, Riley RD. Individual participant data meta-analysis for a binary outcome: one-stage or two-stage? PLoS One. 2013;8: e60650.

35.	Higgins JP, Thompson SG, Deeks JJ, Altman DG. Measuring inconsistency in meta-analyses. BMJ. 2003;327: 557-560.

36.	Thorlund K, Imberger G, Johnston BC, Walsh M, Awad T, Thabane L, et al. Evolution of heterogeneity (I2) estimates and their 95% confidence intervals in large meta-analyses. PLoS One. 2012;7: e39471.

47.	Lambers DS, Clark KE. The maternal and fetal physiologic effects of nicotine. Semin Perinatol. 1996;20: 115-126.

48.	Ion R, Bernal AL. Smoking and Preterm Birth. Reprod Sci. 2015;22: 918-926.

49.	Moore LG. Fetal growth restriction and maternal oxygen transport during high altitude pregnancy. High Alt Med Biol. 2003;4: 141-156.

50.	Koshy G, Delpisheh A, Brabin BJ. Dose response association of pregnancy cigarette smoke exposure, childhood stature, overweight and obesity. Eur J Public Health. 2011;21: 286-291.

51.	Richmond RC, Simpkin AJ, Woodward G, Gaunt TR, Lyttleton O, McArdle WL, et al. Prenatal exposure to maternal smoking and offspring DNA methylation across the lifecourse: findings from the Avon Longitudinal Study of Parents and Children (ALSPAC). Hum Mol Genet. 2015;24: 2201-2217.

52.	Kupers LK, Xu X, Jankipersadsing SA, Vaez A, la Bastide-van Gemert S, Scholtens S, et al. DNA methylation mediates the effect of maternal smoking during pregnancy on birthweight of the offspring. Int J Epidemiol. 2015;44: 1224-1237.

53.	Morales E, Vilahur N, Salas LA, Motta V, Fernandez MF, Murcia M, et al. Genome-wide DNA methylation study in human placenta identifies novel loci associated with maternal smoking during pregnancy. Int J Epidemiol. 2016;45: 1644-1655.

54.	Mattsson K, Kallen K, Rignell-Hydbom A, Lindh CH, Jonsson BA, Gustafsson P, et al. Cotinine Validation of Self-Reported Smoking During Pregnancy in the Swedish Medical Birth Register. Nicotine Tob Res. 2016;18: 79-83.

57.	Groenwold RH, White IR, Donders AR, Carpenter JR, Altman DG, Moons KG. Missing covariate data in clinical research: when and when not to use the missing-indicator method for analysis. CMAJ. 2012;184: 1265-1269.
