# Supplementary material for: Changes in parental smoking during pregnancy and risks of adverse birth outcomes and childhood overweight in Europe and North America: An individual participant data meta-analysis of 229,000 singleton births
Source: PLoS Med. 2020 Aug 18;17(8):e1003182. doi: 10.1371/journal.pmed.1003182 (PMC7433860; doi:10.1371/journal.pmed.1003182)
Supplement: Attachment — Submitted filename: Responses to production issues.docx [file pmed.1003182.s016.docx]

**Responses to production issues**

1. Figure 2-4 are a multipanel figure. Multi-panel figures (those with parts A, B, C, and D) should be submitted as a single file that contains all parts of the figure. Label the figure itself with capital letters, Arial bold font, 12 points.

***Response***: *We adjusted figure 2-4 to multipanel figures. The figures itself are labeled with capital letters in Arial bold font. Because the image is rather large, the size was adjusted to the font size of the figures. We therefore hope that this is about 12 points in print.*

2. Figure 1 has been submitted in a layered TIF file format which we don’t accept for Figures. To ensure that your Figures publish properly, please flatten the layers in each Figure file (Layer→Flatten Image in Photoshop, Image→Flatten Image in GIMP) and re-upload. Make sure to save your TIF files with LZW Compression turned on.
***Response****:* *We merged all the layers. Unfortunately, we do not have Photoshop in our institution, so we hope this is the same as flattening the image. All our TIF files are compressed by using LZW.*

3. Please spell out "odds ratio" at first use in the abstract (line 15).
***Response****: We changed this as suggested. Two small edits were additionally made to keep the word count of abstract within the required 500 words.*

4. Please quote exact p values or p<0.001 in the abstract (e.g., at lines 16 and 17) and throughout the paper.

***Response****: We changed this as suggested.*

5. Can "Tau2=0.00" in some of the figures be avoided by quoting additional decimal places?

***Response****: If Tau2 was 0.00, we now changed this to “Tau2<0.001”.*

6. Please ensure that all Supporting Information files use the following format verbatim throughout the manuscript (including legends and all in-text citations): S1 Fig, S1 Table, S1 Text, S1 Data, etc. Each file type should start at 1 and increase numerically. Please note, however, that the file names themselves must use an underscore rather than a space, i.e. S1_Fig.tif, S1_Table.xlsx, S1_Text.doc, S1_Data.xlsx, S1_PRISMA_Checklist.docx, etc.
***Response****: We have checked and made the appropriate changes when needed.*
